# Impacts of long-term climate change on human health: a global scoping review of quantitative research

**DOI:** 10.1136/bmjopen-2025-108532

**Published:** 2026-09-30

**Authors:** Jesse Limaheluw, Rebecca Niese, Dieneke Bijkerk-Schram, Lisbeth F Hall, Jelske De Kraker, Saskia C J Oostdijk, Fábio Serafim, Ana Maria de Roda Husman

**Affiliations:** 1 National Institute for Public Health and the Environment, Bilthoven, Netherlands; 2 Utrecht University Institute for Risk Assessment Sciences, Utrecht, Netherlands

**Keywords:** Systematic Review, Climate Change, PUBLIC HEALTH

## Abstract

**Abstract:**

**Objectives:**

Research output on the impacts of climate change and human health is growing rapidly. We aimed to synthesise peer-reviewed studies on this subject published between January 2000 and May 2024.

**Design:**

Scoping review.

**Data sources:**

Literature was collected from PubMed, Embase and Scopus on 11 November 2019, 25 June 2021 and 22 April 2024.

**Eligibility criteria:**

We included English language quantitative peer-reviewed primary research articles about the impacts of long-term climate change (ie, as opposed to effects of weather or single climatic events with the exception of attribution studies) on human health.

**Data extraction and synthesis:**

Two reviewers independently screened titles and abstracts. Full-texts were screened by single reviewers, although 23% of publications selected for full-text screening received an independent second-screening. Reported exposures and health outcomes were classified. Reported quantitative effects of long-term climate change on human health were coded as increasing, decreasing, variable or non-significant.

**Results:**

From 60 781 initial records we included 1223 quantitative publications in this scoping review. Health impacts of long-term climate change were studied most often in Europe, the Americas and the Western Pacific. Studies on temperature and on disease vectors, including the diseases they transmit, represented 39.3% and 30.9% of all included publications, respectively. Included results covered over 100 specific health outcomes. Diarrhoeal diseases, malaria, dengue, ischaemic heart disease and stroke were the most studied specific health outcomes. While infectious diseases were often studied in low- and middle-income countries, non-communicable diseases were rarely examined in this context. About 20% of included publications provided retrospective results, demonstrating that climate change is already impacting human health in variable ways globally. About 80% of included publications provided prospective results, projecting mostly variable or increasing impacts on human health over the coming decades.

**Conclusions:**

Our scoping review provides a comprehensive overview of existing evidence about the impacts of long-term climate change on human health. Currently, differences in research methodologies make it difficult to directly compare study findings. The outcomes of this review can help to harmonise future climate change and health research and support evidence-based climate change adaptation.

**STRENGTHS AND LIMITATIONS OF THIS STUDY**

- This systematic scoping review used a comprehensive search to identify quantitative peer-reviewed scientific articles published in English about the impacts of long-term climate change on human health published between January 2000 and April 2024.
- After review of over 60 000 initial records, we extracted descriptive information and quantitative study outcomes from 1223 publications, providing key thematic and methodological research insights and gaps.
- We provide a dataset that can be used to quickly identify and access quantitative evidence on the human health impacts of climate change for any region and country, and over 500 different exposures and 100 different health outcomes included in our review.
- Methodological differences between included publications made it difficult to directly compare study outcomes.

## Introduction

 Climate change impacts human health in many different ways.^1^ These health impacts result directly from changes in exposure to heat or cold, and extreme weather events like wildfires and floods. They also occur indirectly through effects on air, water or food quality, pathogens and disease vectors such as mosquitoes. Implementing existing policies and other commitments to limit climate change will not be enough to keep global warming to less than 2°C by 2100, the goal agreed to by the global community in the 2015 Paris Agreement.^2^ Quantitatively understanding the effects of (continued) climate change on human health is essential for effective climate change adaptation and to reduce future health impacts. Research output on these impacts has grown substantially over the past decades.^3^ To make this increasing amount of information more accessible, and to make it easier to use this information for research, policy, and practice, we present a global scoping review on the impacts of long-term climate change and human health. We specifically aimed to synthesise quantitative studies on the impacts, both observed and projected, of long-term anthropogenic climate change (the long-term and structural shifts in temperatures and weather patterns caused by human emissions of greenhouse gases). In doing so, we also identified research trends and gaps.

## Methods

### Search strategy and selection criteria

Literature was collected from scientific databases Embase (embase.com), MEDLINE (through PubMed) and Scopus (scopus.com) using a broad search query (online supplemental appendix A) with two main concepts: climate change (“climate change”, “greenhouse effect”, “global warming”, “climate variability”, “climate sensitivity”) and health (“health”, “wellbeing”, “mortality”, “morbidity”, “disease”). Using this query, a first search was conducted on 11 November 2019, a second search on 25 June 2021, and a third search on 22 April 2024. We analysed peer-reviewed publications published between January 2000 and May 2024. January 2000 was chosen as a pragmatic cut-off point, allowing us to identify trends in the research field, while not having to include older studies that likely offered outdated results. To be included a publication had to (1) be a full primary research article that was published in English, (2) be about human health, (3) be about long-term anthropogenic climate change (ie, as opposed to effects of weather, or single climatic events with the exception of attribution studies), (4) present quantitative outcomes (eg, percentage change in a number of disease cases) examining impacts of long-term climate change on human health and (5) examine primary or secondary health exposures or effects. These criteria were established at the start of the study and did not change over time. Only English language articles were considered due to language limitations of the authors.

Primary health effects were defined as direct human health impacts caused by climate change. For example, increasing average temperatures directly lead to an increase in heat exposure and associated health outcomes. Secondary impacts were defined as those that arise indirectly through ecological or other environmental changes. For example, changes in temperature and precipitation can lead to changes in microbial water quality which can subsequently cause human health impacts in case of exposure.^4^ Tertiary effects were defined as those resulting from more diffuse and unpredictable interactions between climate and political and socioecological systems.^4^ Publications exclusively on tertiary effects were excluded. The uncertainty associated with these effects was considered too large for the quantitative focus of this study. As such, included outcomes were either direct impacts or underlying hazards, exposures or risks. All study designs, populations and geographical locations were accepted. Articles for which the full text could not be obtained were excluded.

### Screening and data extraction

Duplicate publications were removed in EndNote using the same procedure after each search round.^5^ Titles and abstracts were independently screened by two reviewers (JL and FS for the first two searches, JL and SO for the third search). Disagreement was resolved through discussion between these reviewers. Screening of titles and abstracts was conducted in EndNote (first search),^6^ Covidence (second search)^7^ and ASReview (third search).^8^ The change in tools was needed to accommodate the increased workload following updates to include newer literature. ASReview is an artificial intelligence (AI) supported tool, described in detail elsewhere.^8^ It ranks publications based on relevance using machine learning models which are continuously trained with reviewer decisions as input. For the records obtained through our third search we decided to stop screening after having screened the titles and abstracts of at least 25% of these records, and when at least 100 consecutive papers were excluded.^8 9^ Remaining titles and abstracts were not screened. A different random selection of articles was labelled by both reviewers to start training the machine learning model. We only used ASReview’s default settings. ASReview’s output figures are provided in the online supplemental materials, appendix D. All titles and abstracts were screened for the first two searches. As this was a scoping review, we did not formally assess quality of the included publications.^10^

Full texts were screened once, and data were extracted using an online data extraction form for publications that met the inclusion criteria. All reviewers were briefed on the screening process to ensure consistent interpretation of inclusion criteria and data extraction questions. Publications were grouped by exposure category and assigned to reviewers based on their expertise or personal preference. The different exposure categories used in this study were: air pollution, allergens, extreme events, food, soil, temperature (incl. heat- and coldwaves), ultraviolet radiation (UV), vectors and other animals, water and other (table 1). These categories were defined prior to starting the review. Reviewers could request a second screening by a different reviewer in case of uncertainty about inclusion. Additionally, about 5% of publications were randomly selected to be double screened to check for consistency, and finally JL screened all publications excluded by a different first reviewer. Overall, 363 publications (23%) were double screened which resulted in 60 conflicts. These were resolved through discussion between the respective reviewers. JL, RN, DB-S, LH, JdK and SO were involved in full text screening with additional contributors listed in the acknowledgements.

**Table 1:** Overview of main and secondary exposure categories and number of included studies per secondary category

Main exposure category	Secondary exposure category	Number of included studies	Example exposure
Air pollution	Chemical	146	Ozone
	Microbial	9	Airborne fungi (eg, *Cladosporium* spp.)
	Other	4	Dust
Allergens	Pollen	50	Birch pollen
	Insects	–	–
	Other	3	Limonene (also placed under air pollution–chemical)
Extreme events	Flooding	32	Different types of floods, such as coastal or pluvial floods, were specified
	Landslides	3	–
	Storm	14	Hurricane
	Wildfire	29	–
	Other	5	Avalanche
Food	Chemical	8	Polycyclic aromatic hydrocarbons (eg, Benzo(a)pyrene)
	Microbial	20	Mycotoxins (eg, aflatoxin, also placed under food–chemical)
Soil	Microbial	9	Fungi in soil (eg, *Coccidioides* spp.)
	Other	1	Polycyclic aromatic hydrocarbons
Temperature (incl. heat- and coldwaves)	Cold	87	Physiological temperature stress indicators (eg, wet-bulb temperature)
	Heat	272	
	Coldwaves	13	Different definitions of heatwaves and coldwaves were used, eg, based on duration and intensity.
	Heatwaves	136	
Ultraviolet radiation	–	6	–
Vectors and other animals	Fly (other than mosquito)	28	Sandfly (eg, *Phlebotomus* spp.)
	Mosquito	232	*Aedes spp*.
	Tick	80	*Ixodes spp*.
	Venomous animals	16	Venomous snakes (eg, *Naja spp*.)
	Other	29	Triatomine
Water	Chemical	6	Disinfection byproducts (eg, trihalomethanes)
	Microbial	65	Waterborne pathogens (eg, *Cryptosporidium spp*.)
	Other	–	–
Other	–	15	Adverse driving conditions
Unspecified	–	43	Used, for example, when diarrhoeal diseases were studied without specification of the transmission route

Categories were not exclusive, for example, changes in wildfire-associated emissions of particulate matter were placed under both air pollution—chemical and extreme events—wildfire. Publications studying multiple exposures (eg, heat and cold) were counted for all studied exposures.

### Analysis

Reviewers extracted general study characteristics, methodological information and quantitative results from all included publications. Studies were assigned to one or more WHO regions based on countries covered, and one or more exposure categories (table 1) if applicable. We distinguished three main study outcome types: (1) specific health outcomes, (2) generic or aggregate health outcomes (such as deaths) not associated with a specific cause and (3) exposures. Publications reporting on a specific health outcome were also assigned to a health outcome category. Specific health outcomes were classified following Global Burden of Disease (GBD) classifications.^11^ We specified up to GBD level 3. As some publications specified their outcomes further, we also mapped outcomes to International Classification of Diseases 10th revision (ICD-10) codes (2019 version) if possible.^12^ Health outcomes were considered specific if they were specified in the publication up to at least GBD level 2. Studies that quantified outcomes such as environmental suitability for a specific disease were classified as exposure studies. The observed or projected direction of change in an outcome caused by climate change was characterised as increasing, decreasing, non-significant or variable. A study outcome was considered variable when it was observed or projected to change in different directions depending on the region (geographical variability), population group, climate change scenario, time (temporal variability) or other reason such as climate model. R (last used version 4.4.3) was used for data visualisation (script used for visualisations in online supplemental material, appendix E).

### Patient and public involvement

No patients or public were involved in the design, conduct, reporting or dissemination plans of this research.

## Results

### Descriptive characteristics of the included studies

Following duplicate removal 60 781 publications remained. The titles and abstracts of all 36 958 publications collected through the first two searches were screened manually. Approximately 25% of the 23 824 publications retrieved in the third search were screened using ASReview (5964 by reviewer 1 and 5978 by reviewer 2), before the stopping criteria were met. In total, 1223 publications were included in this review after full text screening (figure 1). Details of all included studies are provided in online supplemental appendix B.

**Figure 1:**
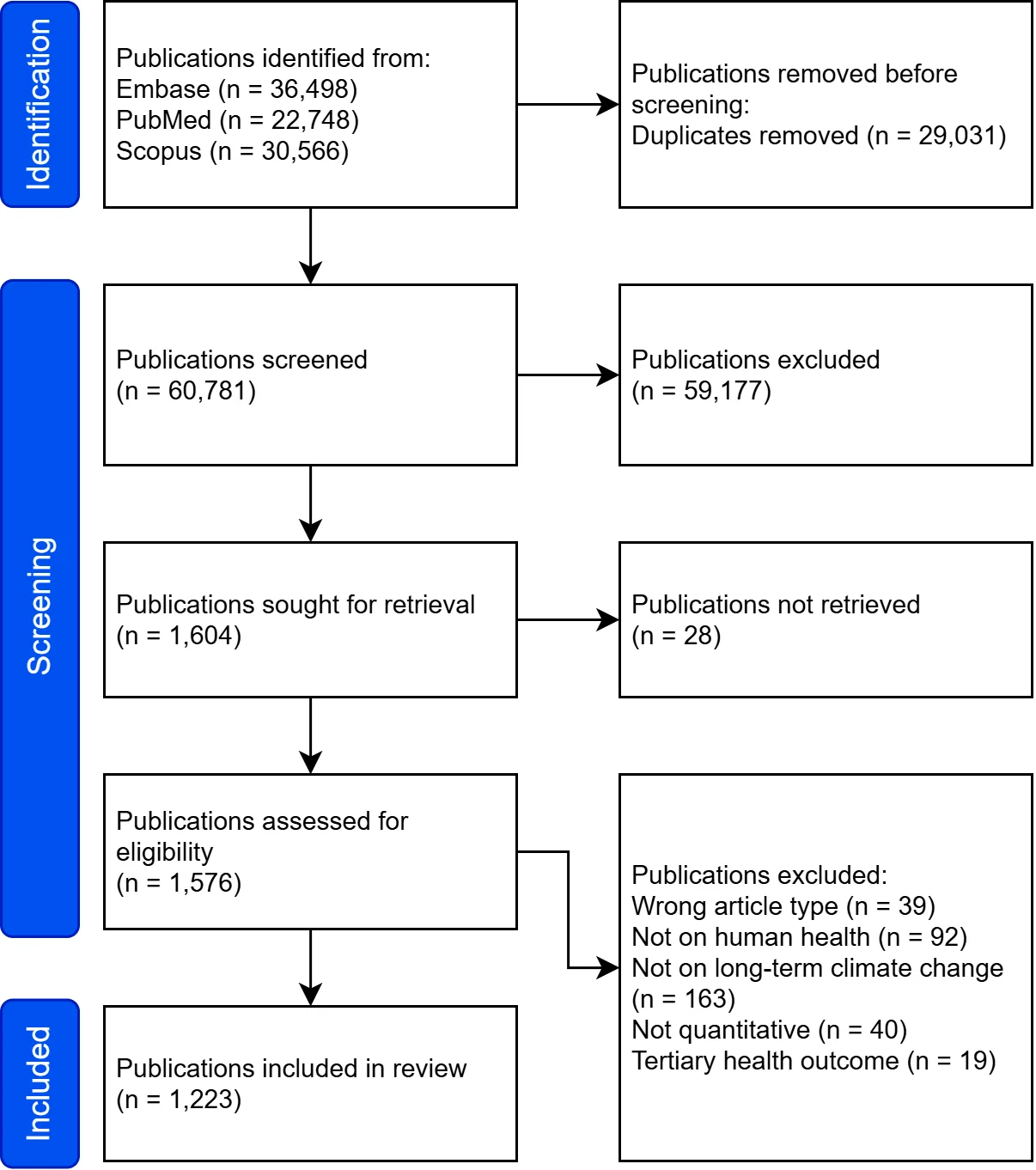
PRISMA flow chart of the review process. The first search retrieved 42 673 publications (published between January 2000 and December 2019), the second retrieved 11 877 publications (December 2019 to June 2021) and the third search retrieved 35 262 (June 2021 to May 2024) publications. PRISMA, Preferred Reporting Items for Systematic Reviews and Meta-Analyses.

The number of included publications per year increased consistently between 2000 and 2024 (figure 2A). We found publications on all WHO regions and 133 studies with global level outcomes. Europe, the Americas and the Western Pacific were the most studied regions overall (figure 2B). The Western Pacific has been the most studied region since 2021. Most publications projected future impacts of climate change (prospective, 1032/1223). Fewer publications examined impacts that have already occurred (retrospective, 254/1223). The time period around 2050 was the best represented in the results (online supplemental materials, appendix C, figure C1). Temperature and vectors have consistently been the most studied exposure categories for all regions and were covered by 481 (39% of total) and 378 (31% of total) publications, respectively.

**Figure 2:**
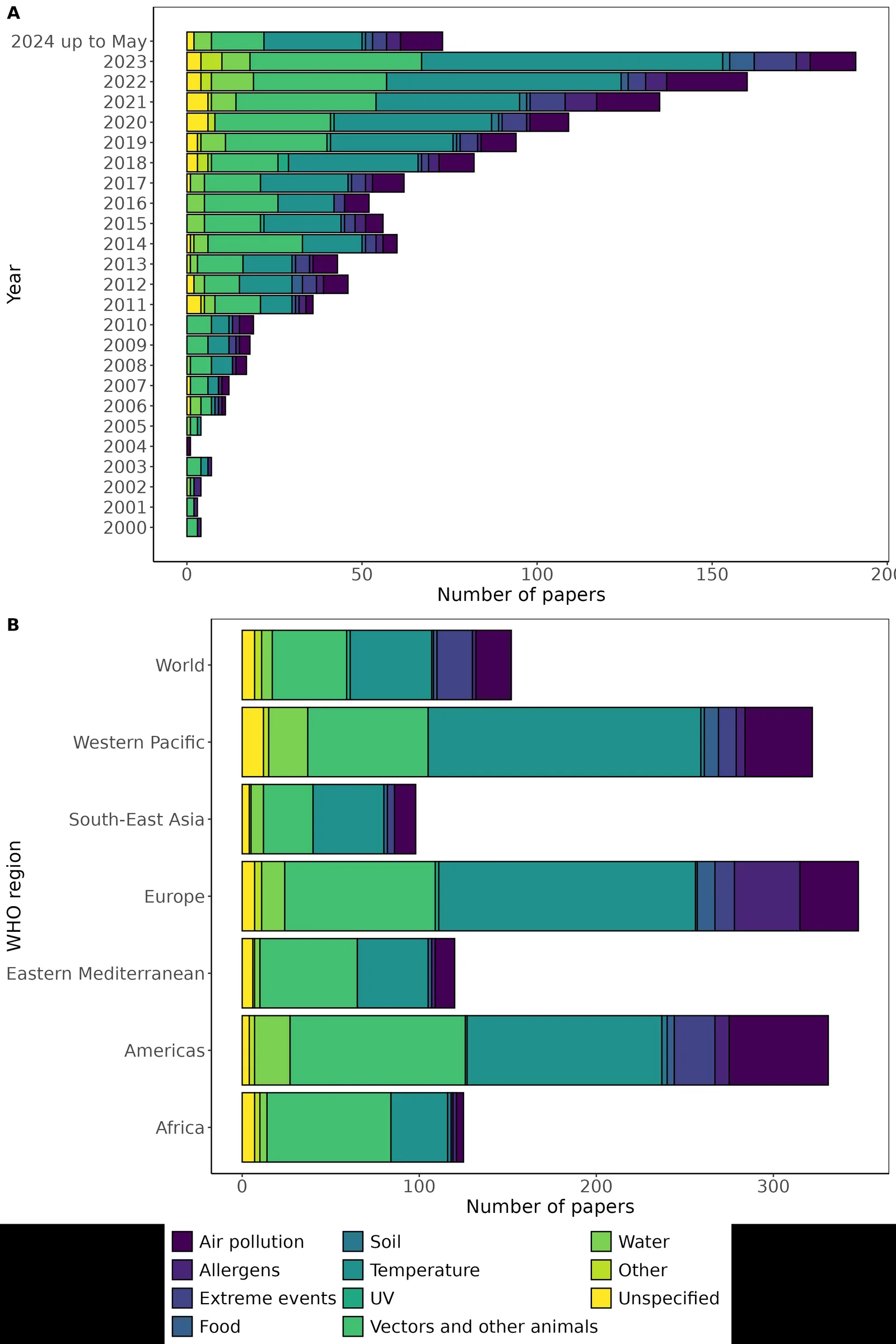
(A) Number of publications per year and exposure category. (B) Number of publications per WHO region and exposure category. A publication was counted only once for each exposure category and WHO region, also when it studied multiple exposures in the same category (eg, multiple air pollutants), or multiple countries in the same region. A paper could be included multiple times for one region if it covered multiple exposure categories. This figure contains data from the 1218 included publications which specified a study region. UV, ultraviolet radiation.

Most studies only quantified impacts on an exposure or a generic health outcome (921/1223, online supplemental materials, appendix C, figure C2). Publications covered 543 specific exposures. Studies on temperature or air pollution often presented the combined impacts on multiple specific health outcomes, such as cardiovascular, cerebrovascular and respiratory diseases, while not presenting results for these outcomes individually.^13–15^

Among the 299 publications that quantified effects on specific health outcomes, we identified results for 72 of 369 specific GBD causes (level 3 and level 2 not specified further), or for 101 distinct outcomes when mapped to ICD-10 codes or code clusters. For 24 additional specific health outcomes publications were included that looked at exposures associated with them, but not at the outcomes themselves (eg, distribution of vectors of a specific disease). 13 out of 72 GBD level 3 causes were studied 10 or more times (in relation to one or more exposures, figure 3). Diarrhoeal diseases were the most studied level 3 outcome, followed by malaria, dengue, ischaemic heart disease and stroke. Additionally, we found over 10 studies for the GBD level 2 causes cardiovascular diseases (not specified further) and chronic respiratory diseases (not specified further).

**Figure 3:**
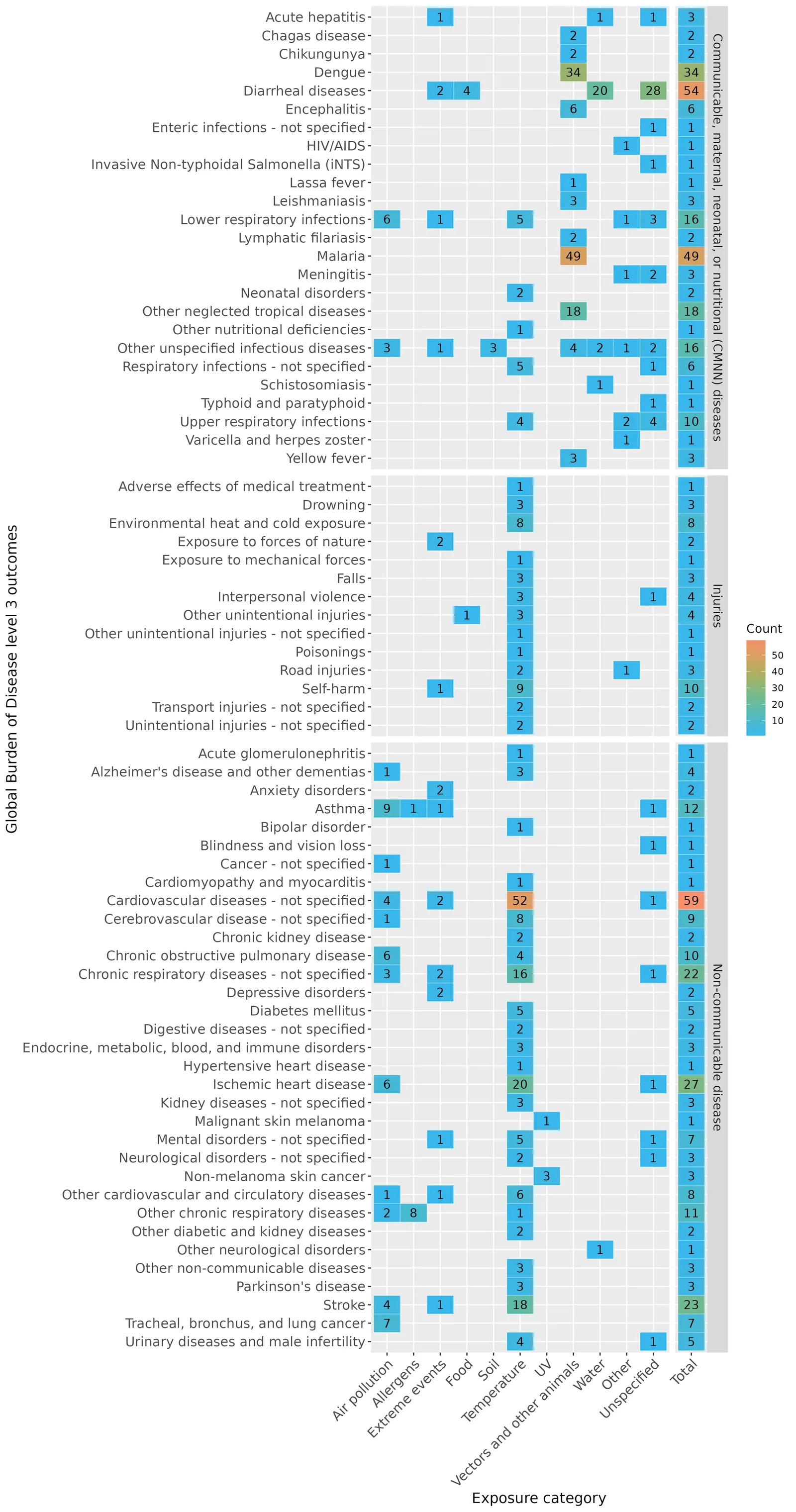
Number of times each Global Burden of Disease (GBD) level 3 outcome or GBD level 2 outcome that was not specified further was studied per exposure category. The last column shows the total number of publications per outcome. If an outcome was associated with multiple exposure categories (eg, health effects of wildfire-related air pollution, which is placed under both air pollution and extreme events) it was counted for all associated categories. This figure does not include studies that quantified effects on an exposure associated with a specific health outcome while not quantifying the health outcome itself (eg, a study that examined the distribution of a vector that primarily transmits a specific disease). This figure contains data from 299 publications that quantified effects on specific health outcomes. UV, ultraviolet radiation.

### Observed impacts of climate change in retrospective research

From 254 publications with retrospective results, we extracted 490 climate change impact outcomes (ie, all individual exposure and health outcomes combined, figure 4). We found publications examining the observed impacts of long-term climate change for all health exposure categories except for food and soil. Of all 490 extracted individual impact outcomes, 52% (256) showed variable change, 35% (171) increased, 9% (44) decreased and 4% (19) showed no (significant) change.

**Figure 4:**
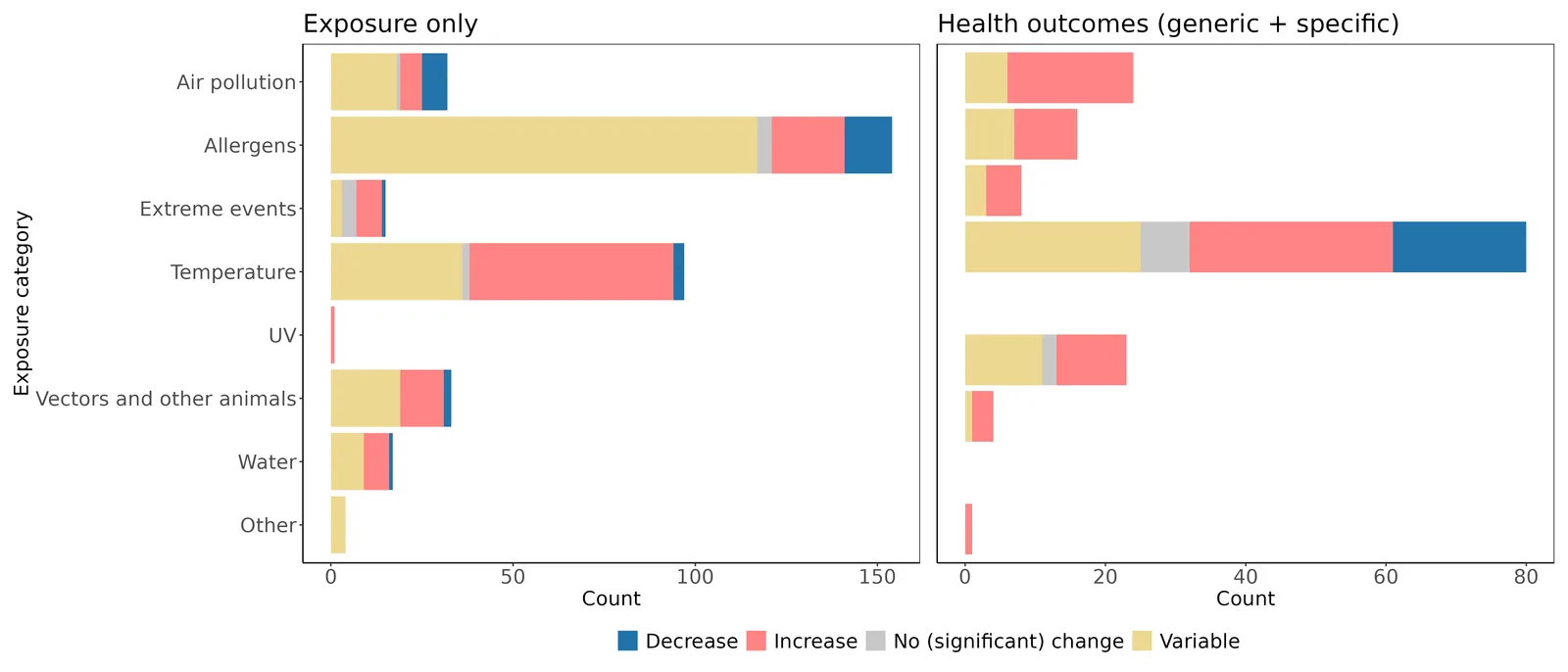
Retrospective climate change impact outcomes from 254 retrospective publications. If a publication examined multiple outcomes, the results for all outcomes are represented in this figure. No retrospective results for the food and soil exposure categories were identified. UV = ultraviolet radiation.

Most included retrospective publications only presented results on exposures (161/254), or generic outcomes (50/254). Most impact outcomes were extracted for allergens. Studies about pollen typically reported impacts on pollen from multiple plant species. Studies on heat or heatwaves showed consistent increases in exposure over time. Variability in study outcomes was mostly due to geographical differences or differences in plant species for studies on pollen. As such, variable results were not necessarily inconclusive. For example, spatial variability in climate change combined with pollutant-specific responses to these changes were found to have driven regional differences in the direction of air pollutant concentration trends across Brazil.^16^ While the overall outcomes were classified as variable, regional outcomes showed consistent increases or decreases.

When considering all health outcomes (figure 4), for the exposure categories air pollution, allergens, extreme events, temperature and water, a majority of publications reported consistently increasing health impacts. Consistently decreasing impacts were only reported for temperature-related outcomes. These decreases were mostly attributed to decreasing cold exposure. Some publications also reported on the possibility of adaptation leading to decreased vulnerability to heat.^17 18^ For example, while the frequency and intensity of ‘hot spells’ in the Czech Republic was found to have increased between 1986 and 2009, a decrease in heat-related all-cause and cardiovascular disease mortality was observed over the same time period for all age groups. The authors hypothesised adaptation, in combination with general quality of life improvements due to socioeconomic development may underlie this trend.^17^ However, these effects were not quantified.

47 retrospective publications presented results on specific health outcomes covering 20 GBD causes (including chronic respiratory, cardiovascular and kidney diseases not specified beyond GBD level 2). For malaria,^19–22^ infections caused by non-cholera *Vibrio* bacteria,^23–25^ Lyme disease^26 27^ and tickborne encephalitis^28 29^ multiple publications reported consistently increasing impacts. Reported variability for all exposure categories was often geographical but could also be due to other factors, such as differences between population groups. For example, one study found that heat-related cardiovascular disease outcomes in Spain only increased for the population over 75 years, while no significant change was observed for other age groups.^30^

For retrospective research different methodological approaches were identified. Observational studies examined trends in outcomes together with long-term climatic trends. A more recent approach is attribution, which quantifies how much of (a trend in) an exposure or health outcome can be attributed to climate change. We included 23 attribution studies. Unlike observational study designs, attribution studies offer direct evidence of climate change as the cause of an observed impact. Attribution is a modelling approach in which outcomes are simulated under scenarios with and without anthropogenic climate forcings (such as greenhouse gas emissions) included. The difference between simulation outcomes then represents the effect of climate change. This approach was applied to examine how climate change altered the magnitude or impact of specific climatic events. For example, one study found that climate change increased heat-related mortality risk by 20% in London and by 70% in Paris during the 2003 heatwave, leading to 64 and 506 additional deaths, respectively.^31^ Long-term trends were also examined with this approach. Several studies on air pollution found evidence of increases or decreases in air pollutant concentrations attributable to climate change.^16 32–35^ For example, one study found changes in ozone concentrations in different countries in East Asia attributable to climate change caused 5600 additional ozone-related deaths between 1980 and 2010.^33^ A publication on summer burned area in California estimated a 172% higher burned area increase between 1971 and 2021 compared with simulations without anthropogenic climate change,^36^ explaining most of the observed changes during this period. One study on pollen in the United States estimated that climate change explained 35%–66% of the advance in the start of the pollen season between 1990 and 2018, but only 4%–8% of the observed increase in total annual pollen concentration.^37^

### Projected impacts of climate change in prospective research

From 1032 publications with prospective results, we extracted 1814 climate change impact outcomes (figure 5). Over two-thirds of all prospective studies only reported on exposures (548/1032) or generic health outcomes (241/1032). Of all extracted outcomes, 52% (937) showed variable change, 40% (733) increased, 6% (114) decreased and 2% (30) showed no (significant) change. Compared with the retrospective publications, we found fewer outcomes on allergens (pollen) and more on all other exposures. Cold-related impacts were consistently projected to decrease. Heat-related impacts were mostly projected to increase. Studies on vectors sometimes considered multiple vector species, explaining the high number of extracted impact outcomes. These studies largely projected variable impacts and indicated that the effect of future climate change on vectorborne disease risks will be highly location-specific and vector-specific. Increasing temperatures and changes in precipitation will expand the area suitable for transmission of vector-borne diseases such as malaria or Lyme, or their vectors, in many regions.^38–41^ However, climate change can also lead to more frequent exceedances of heat or drought thresholds in other regions, creating a less suitable environment.^42^ This dynamic can lead to shifts in disease risks, for example, from lower to higher altitudes, as was projected for malaria in Eastern Africa.^43 44^

**Figure 5:**
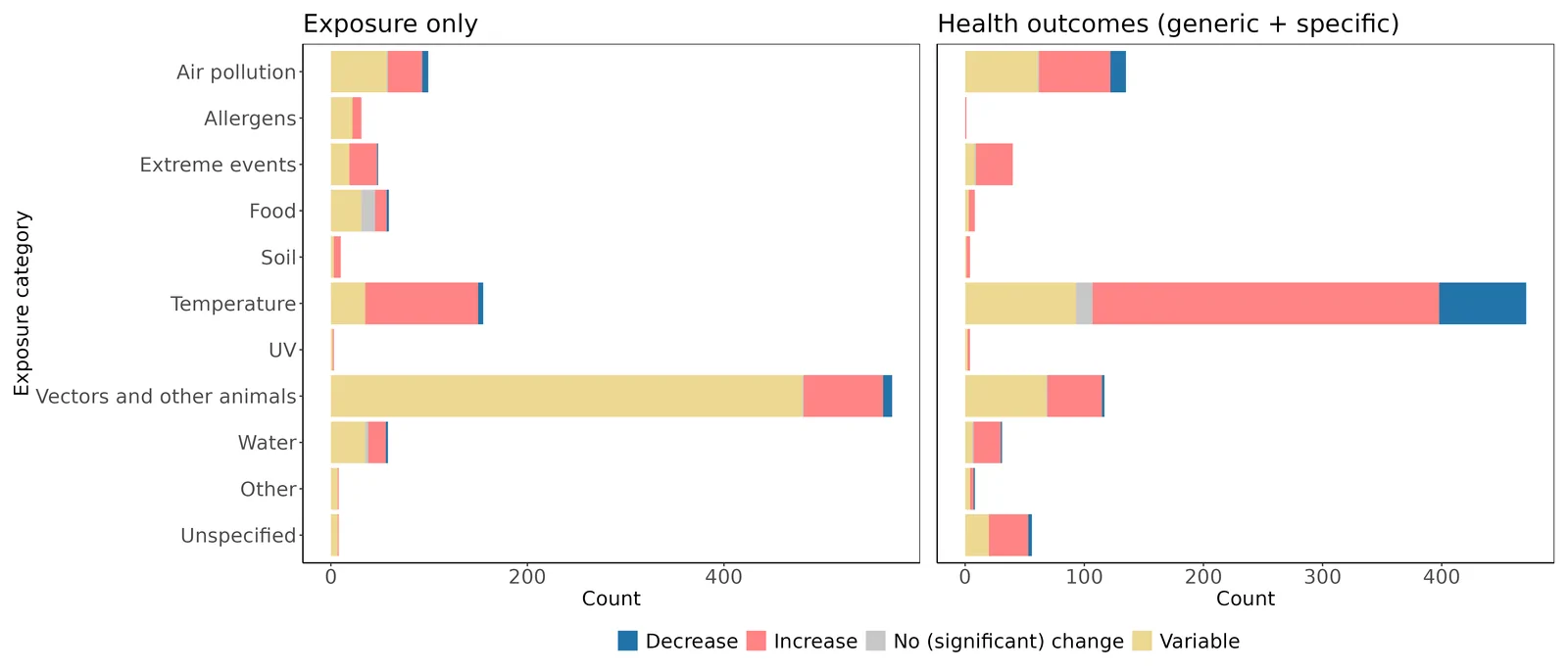
Projected impacts of climate change from 1032 prospective studies on exposures or generic and specific health outcomes. If a publication examined multiple outcomes, all outcomes are represented in this figure. UV, ultraviolet radiation.

Prospective studies quantifying specific health impacts examined 70 specific GBD causes. Except for one publication on congenital heart defects^45^ and one on childhood anaemia,^46^ all publications in the category ‘communicable, maternal, neonatal and nutritional diseases’ examined infectious disease outcomes. Malaria and dengue, both mosquitoborne diseases, were the only specific infectious diseases for which more than 10 prospective publications were identified. Over 10 publications were also included for each of the GBD outcomes diarrhoeal diseases, upper and lower respiratory infections, ‘other neglected tropical diseases’ and ‘other unspecified infectious diseases’, but these can be broken down further based on ICD-10 codes. Diarrhoeal diseases were mostly projected to increase (63% of outcomes), although for some specific diseases, such as rotavirus infections, decreases were also projected.^47 48^ Studies accounting for developments such as economic growth typically found that these developments would lead to a decrease in the overall disease burden of infectious diseases such as diarrhoeal disease or malaria, in line with retrospective trends.^49–51^ However, climate change could slow down this progress. For example, one study projected the possible decrease in diarrhoeal diseases and malaria between 2008 and 2030 without and with climate change, finding a 6.2%–8.9% smaller decrease in the disease burden of diarrhoeal diseases and a 21.2%–27.8% smaller decrease for malaria under various emission scenarios (RCP2.6-RCP8.5).^49^

Studies on non-communicable disease (NCD) and injury outcomes examined 32 specific GBD outcomes. These were mostly associated with temperature and air pollution. Of studies on specific health outcomes related to heat(waves) or to chemical air pollution 83% and 55% projected increases, respectively. We included 10 or more publications on asthma, chronic obstructive pulmonary disease, ischaemic heart disease, stroke, and the level 2 outcomes cardiovascular diseases and chronic respiratory diseases that were not specified further. Outside of the American, European or Western Pacific regions we included only five prospective studies on NCDs^52–56^ and two on injuries.^57 58^ Additionally we included three studies on venomous snakes^59–61^ and two on venomous scorpions^62 63^ (which are associated with specific injury outcomes) for the Eastern Mediterranean and African regions.

A key consideration for prospective research is how to represent future climate change. To account for uncertainty, (combinations of) climate change scenarios are used. Before 2015, most studies used climate change projections based on the emission scenarios in the Intergovernmental Panel on Climate Change’s (IPCC) Special Report on Emission Scenarios. Later we first observed a switch to the Representative Concentration Pathways (RCPs), and then the Shared Socioeconomic Pathways (SSPs) with an associated level of radiative forcing. Included prospective studies examined impacts across all scenarios considered in the IPCC’s assessment reports. Considering the most recent scenarios, this represents levels of global warming ranging between 1.5°C with limited overshoot (SSP1-1.9) to over 4°C (SSP5-8.5).^64^ The most commonly used scenarios were RCP8.5 (or SSP5-8.5, 612/1032 prospective studies, 59%) and RCP4.5 (or SSP2-4.5, 493/1032 prospective studies, 48%), which represent high and intermediate levels of warming respectively. These were also the most common choices for studies using a single scenario, and the most commonly used scenarios in combinations (440/1032 studies, 43%) (figure 6).

**Figure 6:**
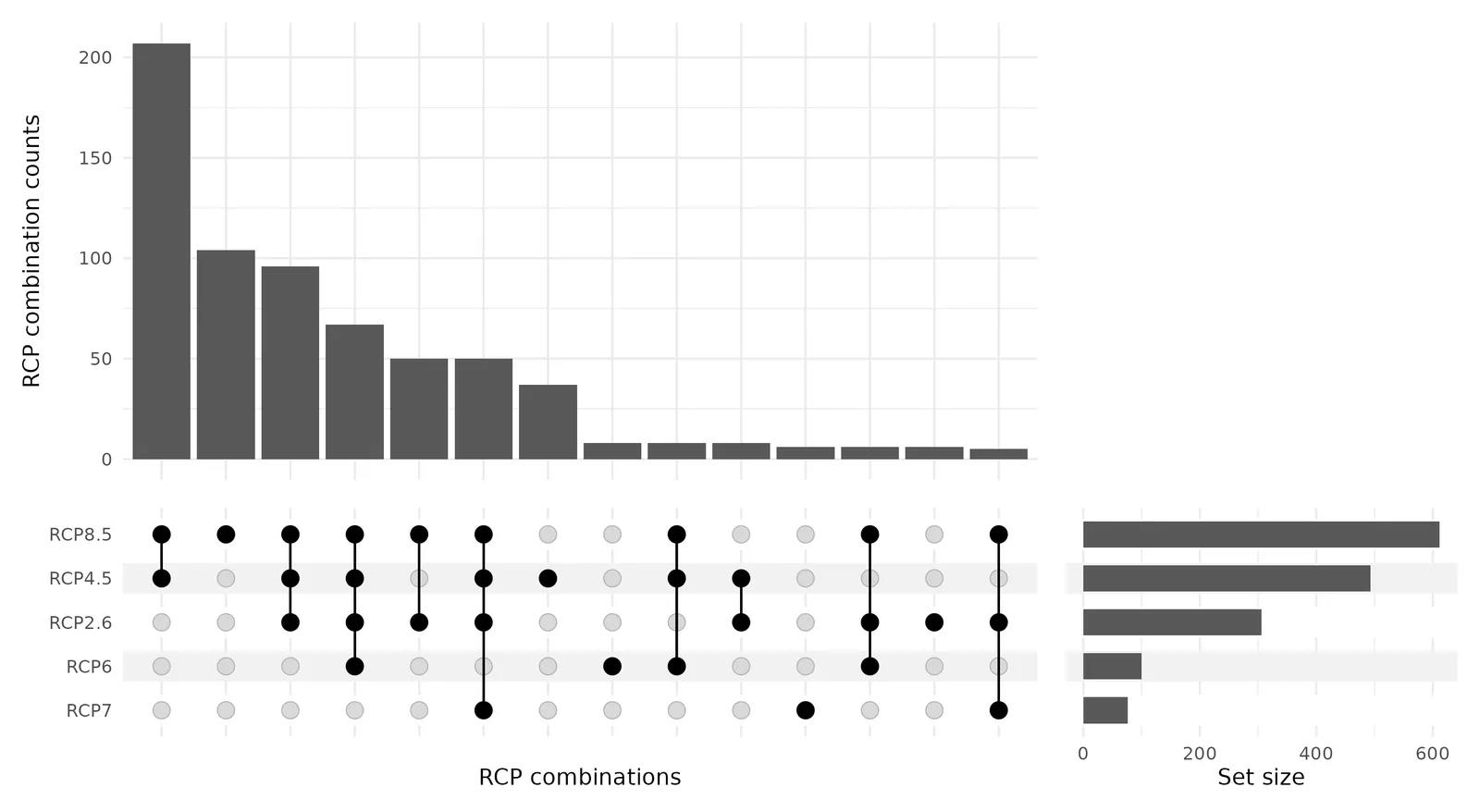
*UpSet plot showing the most commonly used RCP (including SSPs) combinations. Only combinations occurring more than five times are presented in this figure. Set size indicates how often individual RCPs were used. This figure contains data from 692 publications that reported use of RCPs or SSPs*. RCPs, representative concentration pathways; SSPs, shared socioeconomic pathways.

Emission scenarios and associated levels of radiative forcing are used as input for climate models to produce climate change scenarios. Most prospective publications used climate change projections produced by models that are part of the Coupled Model Intercomparison Project (CMIP).^65^ Studies used both individual models or combinations of models (ensembles). Individual models can produce diverging results. For example, one study compared estimates of future global premature deaths due to particulate matter for seven different climate models (GFDL-AM3, MIROC-CHEM, GISS-E2-R, NCAR-CAM3.5, HadGEM2, CAM4 and CAM) and found that average estimates for individual models ranged between −249 976 deaths per year to +1 043 850 million deaths per year by 2100. The ensemble outcome was an increase of 194 448 deaths per year.^66^

## Discussion

Research output on climate change and human health is substantial and growing rapidly. But we identified relatively few studies that provided quantitative evidence on the human health impacts of long-term climate change. After reviewing nearly 61 000 articles published since 2000, we included 1223 peer-reviewed publications that quantitatively examined these impacts. By extracting detailed (quantitative) data from each included publication (online supplemental appendix B) we identified research trends and gaps, aiming to make the great amounts of information presented in these studies more accessible.

Retrospective publications included in our review largely showed that climate change already affects human health globally. However, the number of included retrospective publications was low compared with the number of prospective publications. We included retrospective studies on 20 GBD outcomes, which largely overlapped with the outcomes highlighted in the IPCC’s latest Working Group 2 report as ‘sensitive to climate change’.^1^ Unlike previous reviews on climate change and health,^3^ our study did not consider short-term associations with weather or the impacts of individual climatic events such as a specific hurricane. While historical associations between meteorological factors and health outcomes can inform projections under future climate scenarios, they do not necessarily provide evidence of actual climate change impacts. Our work offers a clearer picture of the evidence specifically focused on the impacts of long-term climate change.

Prospective research showed that many health outcomes could be affected by climate change in the future. However, many outcomes for which we identified research were represented by single studies. A key knowledge gap is the lack of evidence on NCDs in most (often low- and middle-income) countries in the African, Eastern Mediterranean and South-East Asian regions. In many of these countries NCDs already make up a large share of the overall disease burden, and insights from other regions show that these health outcomes are sensitive to climate change. However, as there are often large differences between lower and higher income regions in terms of demographics, access to healthcare or the quality of the living environment, available evidence from other (high-income) countries is not generalisable to these settings. We therefore highlight the urgent need for more research on NCDs and climate change in low- and middle-income regions.

Accounting for other trends, such as urbanisation, population growth, demographic change, (forced) migration or climate change adaptation, can significantly affect estimates of future impacts. Adaptation to heat was shown to decrease heat-related impacts in some retrospective studies, despite increasing exposure. Prospective studies accounting for adaptation indeed found it can (partially) offset additional impacts caused by future climate change.^67–69^ For example, one study on future heat-related mortality in Tianjin, China, incorporated a reduction in heat-related mortality of 25% to account for adaptation.^67^ This was enough to offset the increase in heat-related mortality due to climate change under RCP2.6, but not under RCP4.5 by 2070, or under RCP8.5 by 2050. The level of adaptation required to offset climate change impacts generally increases with higher levels of warming. This may lead to unmanageable situations in settings or populations with limited (additional) capacity to adapt. Studies on air pollution highlighted the influence of climate change mitigation and other environmental policies on human emissions of air pollutants. Some studies showed these measures could compensate for the future impacts of climate change,^70^ but others did not.^71^ For example, one study examining future ozone and particulate matter (PM_2.5_) concentrations in California found that implementing stringent air pollution control and climate change mitigation measures would effectively offset increases in air pollution caused by climate change in 2035. However, current policies were found to be insufficient.^70^ Depending on the pollutant or location of interest, specific processes such as increases in biogenic ozone^72^ or wildfire-associated particulate matter^73^ may be important and outweigh the effect of emission-reducing measures. Generally, most extracted (retrospective and prospective) climate change impact outcomes were coded as variable. This did not always mean that results were inconclusive. More often it meant that we could not assign a clear direction of change to the overall outcomes within our coding framework. For example, results presented at a high spatial resolution almost always showed geographical variability. For effective climate change adaptation, results should be considered with respect to their specific local or regional context if possible.

The use of standard climate change projection pathways and climate models is a good example of harmonisation of research methods. Deciding which scenario(s) to use in an assessment should consider the relevance of different scenarios to policies and practice. We recommend inclusion of scenarios that represent, for example, the temperature targets set by the Paris Agreement or expected warming at different levels of mitigation policy implementation. In the current study, differences in methodologies, outcome measures and reporting of results between the included publications made it impossible to directly compare quantitative outcomes of different studies. This is a major challenge for the practical application of climate change and health research. Being able to make a direct quantitative comparison between outcomes of different studies is useful to effectively prioritise climate change impacts in adaptation policies and actions. One way to produce comparable estimates for different outcomes or regions is to examine them in a single methodological framework.^74 75^

For feasibility reasons, some decisions were made to limit the scope of the study. By including only English-language literature and peer-reviewed research indexed in the used databases, we have likely missed local-level evidence. However, many included studies provided results at a high spatial resolution and may still offer valuable insights at the local level, depending on the resolution of input data, especially for identifying general patterns and needs for targeted local research. If non-peer-reviewed and other grey literature had been included, or more direct quantitative comparisons between extracted results had been done (eg, through meta-analysis), it would have been important to formally assess study quality. This is not typically done when performing a scoping review.^10^ With research output on climate change and health quickly growing, we acknowledge that new relevant publications have become available since collecting the literature for this review. We used the AI-supported review tool ASReview to enable the screening of the many titles and abstracts collected through the third search in a relatively short time frame (compared with the first and second screening rounds). As we only screened about 25% of all titles and abstracts it is possible relevant publications have been missed. However, the share of articles included after full-text screening was consistent (2%) between the first two searches and the third search. Additionally, both reviewers using ASReview had excluded almost 500 consecutive irrelevant papers when both stopping criteria were met. As such, we do not expect any missed papers to have significantly altered our findings. Similarly, it was not feasible to double screen all full texts, and complete double screening may have yielded some additional inclusions, although several steps were taken to ensure and check for consistency between reviewers. To make setting up and maintaining an essential up-to-date knowledge base on climate change and health more feasible, we recommend that future reviews continue to explore the use of machine learning and other novel techniques, as has been demonstrated in other climate change and health studies.^3^ Further harmonisation of research methods and outcome reporting will allow these tools to be used more effectively, including for full-text screening and data extraction.

Another methodological choice made to limit the study scope was the exclusion of tertiary health outcomes. Following Butler and Harley,^4^ such outcomes could include violent conflict or famine, and were extended by us to include for example drought exposure. The impact of these outcomes can be severe, and a link to climate change is present.^1^ However, it is difficult to quantitatively establish the effect or contribution of climate change for these impacts. For example, projecting armed conflict risk under long-term climate change is hindered by several methodological, technical and policy-related challenges.^76^ Practically, primary and secondary outcomes are more suitable targets for climate change and health indicators and policy, as the impact of climate change can often be demonstrated and tracked over time, and because some outcomes may already be covered by existing monitoring programmes and projects.^77^

While many specific health outcomes were identified in the included publications, many more have never been studied in the context of long-term climate change. We can deduce that as exposures like temperature or air pollution will continue to be affected by climate change, any health outcomes influenced by these exposures will be affected as well. Some specific outcomes are more sensitive to changes in exposures than others. For example, based on established sensitivity to fine PM_2.5_ one study found that 42% of the projected change in PM_2.5_-related deaths in China attributable to climate change by 2050 was caused by stroke, while ischaemic heart disease, chronic obstructive pulmonary disease and lung cancer made up smaller shares of the overall change.^78^ For targeted health action, it may be necessary to look beyond general health outcomes such as all-cause deaths, and conduct additional research on specific health outcomes. For infectious diseases, the disease burden of over half of all human infectious diseases can be aggravated by climate change based on retrospective data.^79^ We included studies on 54 specific infectious diseases (based on ICD-10 codes). While these included the most important causes of disease burden in the present, publications on specific diseases with local relevance may be lacking.

Based on the thematic and methodological gaps identified through this review we propose a series of recommendations that can be used to guide and strengthen future climate change and health research:

1. For the prioritisation and focus of future research we recommend:
  - That more consideration should be given to NCDs in lower- and middle-income countries. Evidence on NCDs comes primarily from high-income countries. This evidence may not be generalisable to other settings but does demonstrate that climate change causes and exacerbates NCDs.
  - That more consideration should be given to non-vector-borne infectious diseases. Prioritisation can be based on established climate sensitivity and context-specific relevance (eg, current disease burden, public health preparedness, expected suitability).
  - A focus on specific health outcomes, preferably associated with a specific exposure (if possible and relevant). For example, diarrhoeal diseases were often studied as a single outcome, even though different causes of diarrhoeal disease may require different adaptation approaches. Adaptation efforts will benefit from cause-specific insights.
  - Expanded use of attribution approaches for retrospective studies to clearly demonstrate the current impacts of climate change, if data availability allows it.
  - That regular reviews, preferably every year, on the impacts of long-term climate change on human health should be carried out using a standardised methodology, for example, based on our approach. This will help to maintain an up-to-date knowledge base and an overview of the most pressing research and knowledge gaps. To make this more feasible, we recommend further integration of machine learning tools and other novel methods into the review process.
2. To strengthen projections of future climate change impacts we recommend:
  - Use of multimodel ensembles and a range of climate change scenarios to better account for the inherent uncertainty associated with modelling future climate change.
  - Selection of climate change scenarios that enable comparison of impacts at different policy-relevant levels of global warming, such as the targets set out in the 2015 Paris Agreement (1.5°C–2°C, SSP1-1.9 – SSP1-2.6) and expected warming under emissions trajectories given implementation of current mitigation policies (eg, SSP2-4.5).
  - Integration of other relevant non-climate developments such as changes in demographics, land use, urbanisation, access to healthcare, access to adequate drinking water, sanitation and hygiene (WASH), climate change mitigation and climate change adaptation. Reporting should clarify the specific attribution of climate change.
3. To improve harmonisation of methodologies we recommend:
  - Further harmonisation of outcome metrics, especially for exposure studies. We identified a wide range of different metrics used in exposure studies including for the same exposures. For health outcomes, apart from mortality, more informative metrics such as disability-adjusted life-years (which also aligns with GBD reporting) should be considered.
  - Prospective research should harmonise the choice of emissions/climate change scenarios and climate models. Climate change scenarios should preferably align with scenarios considered by the IPCC’s Working Group 2 (currently the SSPs). Climate data should preferably be produced by models which are part of CMIP. For local or regional assessments, country-specific scenarios may be preferred, but reporting should link the scenarios used to their global companion scenarios.
  - Prospective research should harmonise data inputs on other developments such as demographic change. For example, in line with the previous recommendation, the socio-economic assumptions underlying the SSPs should be applied when using these scenarios.
4. To improve reporting we recommend minimum reporting standards including:
  - Specification of the baseline year and time period (retrospective) or target years (prospective) for which the outcomes are presented.
  - Specification of the used emissions/climate change scenarios and climate models.
  - Clearly defined outcome metrics.
  - Specification of all non-climate input data and their sources.
  - Interpretation of variability in the outcomes.
  - Discussion on the implications of the research for climate change adaptation purposes.

## Conclusions

Implementation of existing climate change mitigation policies and commitments will not be enough to limit global warming to 1.5°C–2°C by the end of this century. Considering this, human health should be an integral part of climate change adaptation efforts.^77^ Besides stepping up general health actions such as poverty reduction and improving access to healthcare, this requires quantitative and accessible knowledge on climate change impacts. Our scoping review provides a comprehensive overview of existing evidence, some of which may hold direct practical value for adaptation, while also offering a starting point for further targeted research. We highlight the knowledge and research gaps on understudied exposures, regions, specific health outcomes and methodological harmonisation.

## Supplementary material

10.1136/bmjopen-2025-108532 online supplemental file 1

## Data Availability

All data relevant to the study are included in the article or uploaded as supplementary information.
