# Supplementary material for: Impacts of long-term climate change on human health: a global scoping review of quantitative research
Source: BMJ Open. 2026 Sep 30;16(9):e108532. doi: 10.1136/bmjopen-2025-108532 (PMC13630005; doi:10.1136/bmjopen-2025-108532)
Supplement: Supplementary file 1 [file bmjopen-2025-108532.reviewer_comments.pdf]

PEER REVIEW HISTORY

BMJ Open publishes all reviews undertaken for accepted manuscripts. Reviewers are asked to complete a checklist review form and are provided with free text boxes to elaborate on their assessment. These free text comments are reproduced below.

ARTICLE DETAILS

Title (Provisional)

Impacts of long-term climate change on human health: a global scoping review of quantitative research

Authors

Limaheluw, Jesse; Niese, Rebecca; Bijkerk-Schram, Dieneke; Hall, Lisbeth; De Kraker, Jelske; Oostdijk, Saskia; Serafim, Fábio; de Roda Husman, Ana Maria

---

VERSION 1 - REVIEW

**Reviewer**	**1**
**Name**	**Fisher, Georgia**
**Affiliation**	**Macquarie University, Australian Institute of Health Innovation, Centre for Healthcare Resilience and Implementation Science**
**Date**	**26-Aug-2025**
**COI**	**None**

Thank you for the opportunity to review this scoping review. The review covers an important topic that is highly relevant to modern medicine, and the authors are to be commended for including such a large volume of literature in their review. While I believe this review covers an essential topic, I do have some concerns about its conduct. These concerns (major and minor) are detailed below. I invite the authors to respond to these and provide clarification or justification where appropriate.

Major concerns / comments

Summary:

Throughout:	The summary could better emphasise the review's focus on the long-term impacts of climate change on human health.

Key messages:

P3, Lines 31-35	The authors state that previous research has not moved "beyond synthesis", and that this is the gap they are addressing in their review. It is unclear what precisely is "beyond synthesis", and how this review achieves this.
P3, Lines 37-41	This section should focus on what the study adds to the literature (i.e., what is novel, that has not been done before – the database), rather than simply detailing the study

methods.

P3, Lines 43-47 — The authors state that their review highlights "best practices", however these are not reported in either the results or significantly detailed in the discussion. The authors could also clarify in what specific area they highlight best practice (e.g., climate projection methodology? scoping review methodology?).

P3, Lines 56-57: — It would be helpful to provide a reference that demonstrates increasing literature on this topic.

There are several reviews highlighting an exponential increase in the literature in this area.

Methods:

P4, Lines 68 – 69 — The authors should provide the rationale for excluding articles before the year 2000.

P4, Line 72 — The authors should be more specific about what they mean by "quantitative outcomes" – e.g., "*quantitative outcomes examining the impact of long-term climate change on human health*"

Throughout: — The authors state that many aspects of this review were pre-determined and did not change over time. However, there is no protocol registered for this review, which makes this constancy very hard to determine. This is a considerable methodological flaw in the review.

P4, Line 74 — Was there a reference for / pre-defined list of what was defined as a primary health impact?

P4, Line 75 — The distinction between a primary and secondary impact is currently slightly unclear. The authors could give a more specific example of each, including the mechanism of impact, to clarify this difference.

P4, Line 83: — Did you exclude results for which data was missing? Please clarify if you excluded or if you contacted authors for missing details.

P5, Line 85 — How were duplicate records removed? Did this differ between searches, as the screening methods did? If so, how did the authors account for duplicates across searches? That is, there may have been records collected in both search 1 and search 2 – if these searches were de-duplicated in separate programs, how were these duplicates picked up?

Could the authors also clarify which authors were involved in which screening steps.

P5, Line 94 — Could the authors please include ASReview's search summary figures as supplementary material (e.g. below)

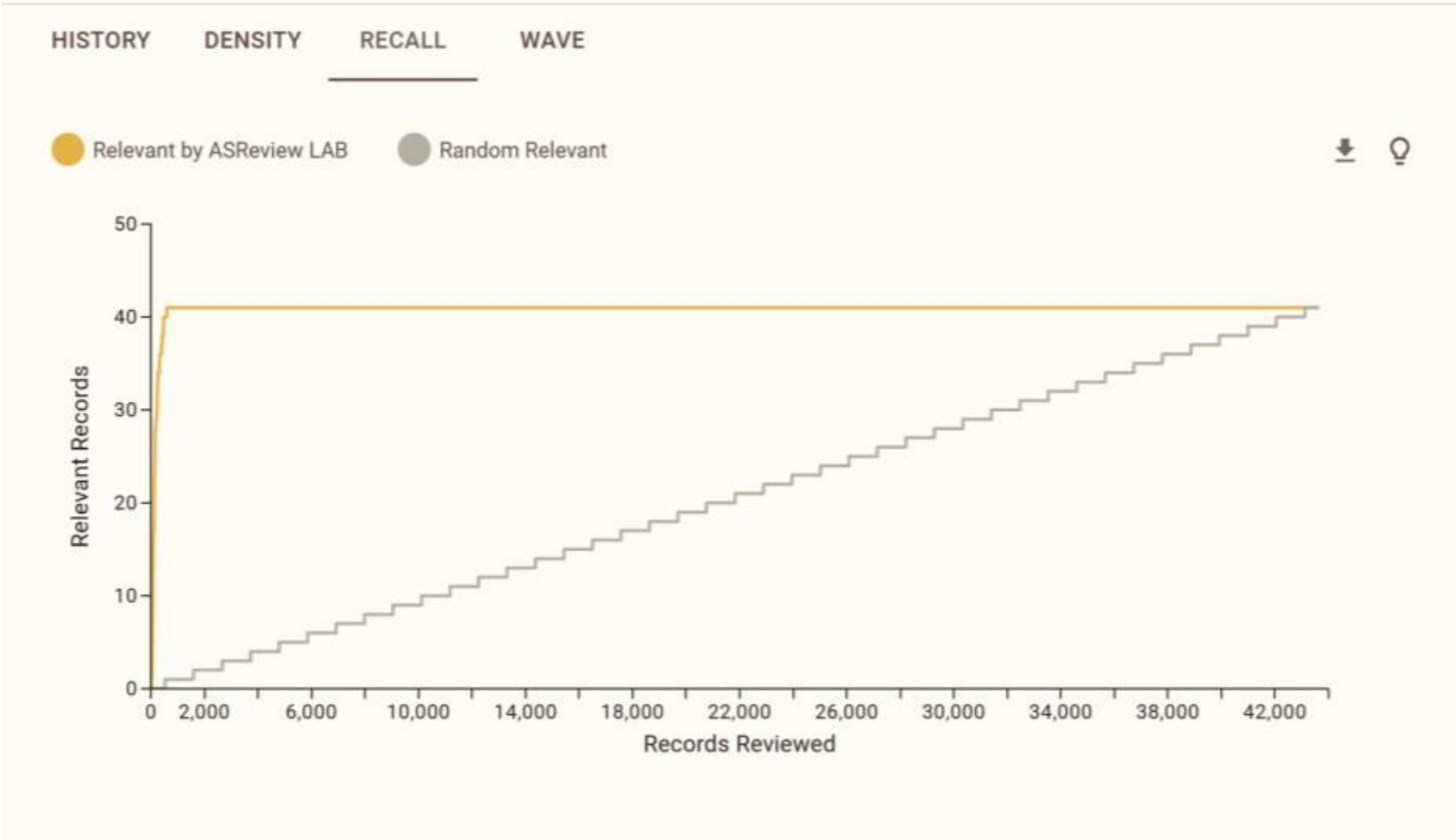

P5, Lines 101-4	How were these exposure categories generated? Did they come from the literature or did the authorship team create them a-priori?
P5, Lines 106-6	While the authors are to be commended for reporting their rate of disagreement, this rate is unusually high (a 28% conflict rate). How many of these were incorrect includes, and how many were incorrect excludes? Assuming all conflicts were incorrect excludes, extrapolating this rate to the large volume of articles screened at the full text stage means that as many as 397 papers are missing from the authors' analysis. This is of major concern.

Results

Throughout results.	The authors should report precise counts throughout their methods where they state<br><br>For example, P8, Line 161 states "*Most studies only quantified impacts on an exposure or generic health outcome*". How many studies? It is also convention to report (n = x, X%) in all results.<br><br>The authors could also provide more concrete examples throughout their results. For example, P8 Lines 162 – 165 states "*Studies on temperature or air pollution often presented the combined impacts on multiple specific health outcomes, such as cardiovascular, cerebrovascular, and respiratory diseases, while not presenting results for these outcomes individually*." An example of one such study, and its result, would be helpful here.
Figures	Each figure legend requires a total n represented in the figure.<br><br>Please specific the program used to create figures and supply the analysis source code so that this can be used in conjunction with the author's dataset to replicate their figures.<br><br>They are difficult to understand in B+W. The authors might consider texture or different colours to compensate for B+W printing.

Discussion

P11, Line 277 – 288	The authors state here that their data was used to identify gaps, however this was not a specific study aim and these gaps are absent from their results.
P12, Lines 299-312	It would be useful to highlight a specific example of how local or regional context altered the impact of adaptation and mitigation.
P12, Lines 313-321	This paragraph details new data that are not reported in the results. Given that this paragraph suggests the authors have additional data, it would have been an interesting addition to the results to understand the emissions projections used in each prospective study, and the modelling / prediction methods used, particularly given that the authors are aiming to describe "best practice" in this dataset.
P 12, Lines 328-30	The authors could suggest a potential single methodological framework, or how one might be selected.

Minor concerns / comments

Summary

P2, Line 14	The authors could better open their summary with "*Research output on the impact of climate change on human health is growing …*" to better reflect the purpose of their review.
P2, lines 18-19	The authors would be better to give precise percentages here.

Introduction

P4, Line 58-59	The authors could instead state that they "*present a global scoping review on the impact of long-term climate change on human health*" to better reflect the purpose of their review.

Methods

P4, Line 76	Suggest changing to "*Secondary impacts <u>were defined as those that</u> arise indirectly"*
P4, Line 78	"*Tertiary effects were defined as <u>those</u> resulting from …*"

Results

P9, Line 195	"*for the exposure categories air pollution, allergens, extreme events, temperature, and water a majority of publications .."* is missing a comma after water.

Discussion

P12, Line 320-21	"*For example, because it is difficult to determine what the biggest (local) impacts of climate change will be and should thus be prioritized in climate and health policies."* – this sentence does not make grammatical sense.
P13, Line 353-4	"*such outcomes could include violent conflict or famine and was extended by us to include for example drought exposure*." – 'was' should be 'were'

**Reviewer**	**2**
**Name**	**Priya, Harsh**
**Affiliation**	**All India Institute of Medical Sciences, Public Health Dentistry**
**Date**	**07-Sep-2025**
**COI**	**None**

Thanks for submitting this excellent manuscript.

It is an extensive study. Quite well presented.

There are few suggestions

The summary says that around 20% of studies looked at past impacts and 80% predicted future impacts. But it doesn't explain how reliable these studies are. Since the review didn't check study quality, this percentage split could be misleading.

Inclusion criteria were unclear, making it hard to distinguish weather changes from long-term climate trends, which may have caused inconsistent study selection.

Using ASReview in methodology section but screening only 25% of papers may be a big issue. Even though it mentions stopping rules, this could create bias, especially for less studied regions or outcomes. The sensitivity analyses is not provided in the manuscript to show how much this choice might affect results.

Only about 11% of articles were double screened. For such a diverse topic and with AI involved; this is too low. Also, inter-rater agreement kappa score is not reported.

The quality or risk of bias of included studies was not assessed. While this is sometimes acceptable in scoping reviews, here it may be a serious limitation because they extracted numerical results. This means poor-quality studies may have influenced findings just as much as stronger studies.

The retrospective results are mostly labeled as "variable," but the authors do not investigate why. Differences in study design, exposure definitions, or regional focus could explain this heterogeneity. Without such analysis, the "variable" category adds little value.

VERSION 1 - AUTHOR RESPONSE

Reviewer 1

Thank you for the opportunity to review this scoping review. The review covers an important topic that is highly relevant to modern medicine, and the authors are to be commended for including such a large volume of literature in their review. While I believe this review covers an essential topic, I do have some concerns about its conduct. These concerns (major and minor) are detailed below. I invite the authors to respond to these and provide clarification or justification where appropriate.

Major concerns

Summary:

Throughout: The summary could better emphasise the review's focus on the long-term impacts of climate change on human health.

We have added some more references to long-term climate change in the summary with an explanation under 'eligibility criteria'.

Key messages:

The key messages section had to be removed as per journal guidelines. Some clarifications offered below.

P3, Lines 31-35 The authors state that previous research has not moved "beyond synthesis", and that this is the gap they are addressing in their review. It is unclear what precisely is "beyond synthesis", and how this review achieves this.

Some previous reviews on the subject focused primarily on research trends (e.g., Berrang-Ford, 2021, which also examined titles and abstracts only). We also wanted to provide insight into and synthesize the actual research based on their full texts.

P3, Lines 37-41 This section should focus on what the study adds to the literature (i.e., what is novel, that has not been done before – the database), rather than simply detailing the study methods.

The new 'strengths and limitations' section had to focus specifically on methodological aspects, and not novelty etc. as per editor instructions.

P3, Lines 43-47 The authors state that their review highlights "best practices", however these are not reported in either the results or significantly detailed in the discussion. The authors could also clarify in what specific area they highlight best practice (e.g., climate projection methodology? scoping review methodology?).

We agree that this should have been given a more prominent position in the text. We now end the discussion with a list of recommendations (thematic and methodological) for future climate change and health research (retrospective and prospective). We have also made some amendments throughout to more clearly support these recommendations.

P3, Lines 56-57: It would be helpful to provide a reference that demonstrates increasing literature on this topic. There are several reviews highlighting an exponential increase in the literature in this area.

We could not add this to the strengths and limitations but we have added reference [3] to the introduction in support of the sentence "Research output on these impacts has grown substantially over the past decades".

Methods:

P4, Lines 68 – 69 The authors should provide the rationale for excluding articles before the year 2000.

Added under Section 2.1: "January 2000 was chosen as a pragmatic cut-off point, allowing us to identify main trends in the research field, while not including older studies that likely offered outdated results."

P4, Line 72 The authors should be more specific about what they mean by "quantitative outcomes" – e.g., "quantitative outcomes examining the impact of long-term climate change on human health"

Clarified as: "4) present quantitative outcomes (e.g., percentage increase in a number of disease

cases) examining impacts of long-term climate change on human health […]"

Throughout: The authors state that many aspects of this review were pre-determined and did not change over time. However, there is no protocol registered for this review, which makes this constancy very hard to determine. This is a considerable methodological flaw in the review.

The review was initiated in 2019, and at the time protocol registration for scoping reviews was not common or possible for main databases such as Prospero. We do agree that protocol registration or publication would have been more methodologically sound. However, protocol registration also does not mean methods cannot change afterwards. Our methodology as presented provides an accurate overview of the methods used in our review, and any changes in methodology over time (such as the changes in screening tools) have been reported.

P4, Line 74 Was there a reference for / pre-defined list of what was defined as a primary health impact?

The cited reference [4] provides the definition for primary, secondary, and tertiary health impacts that we used in our review. This classification was only used to separate primary/secondary outcomes from tertiary outcomes.

P4, Line 75 The distinction between a primary and secondary impact is currently slightly unclear. The authors could give a more specific example of each, including the mechanism of impact, to clarify this difference.

We have expanded the explanation: "Primary health effects were defined as direct human health impacts of caused by climate change. For example, increasing average temperatures directly lead to an increase in heat exposure and associated health outcomes. Secondary impacts arise indirectly through ecological or other environmental changes. For example, changes in temperature and precipitation can lead to changes in microbial water quality which can subsequently caused changes in human health impacts in case of exposure."

P4, Line 83: Did you exclude results for which data was missing? Please clarify if you excluded or if you contacted authors for missing details.

We already state "Articles for which the full text could not be obtained were excluded." We did not contact the authors of these papers. We were able to extract data from all included publications.

P5, Line 85 How were duplicate records removed? Did this differ between searches, as the screening methods did? If so, how did the authors account for duplicates across searches?
That is, there may have been records collected in both search 1 and search 2 – if these searches were de-duplicated in separate programs, how were these duplicates picked up?

We have added a reference for our deduplication procedure, which was the same throughout (De-duplication of database search results for systematic reviews in EndNote - PMC). The screening was done using different tools, but the deduplication was always done in Endnote. We did find some additional duplicates in the final dataset between submission and resubmission (between searches 2 and 3). We have updated all numbers and figures accordingly.

Could the authors also clarify which authors were involved in which screening steps.

We have specified for title and abstract screening: "JL and FS for the first two searches, JL and SO for the third search"

And for full text screening: "JL, RN, DBS, LH, JdK, and SO were involved in full text screening with

additional contributors listed in the acknowledgements."

Author contributions to specific research steps were also detailed in the Contributors section.

P5, Line 94 Could the authors please include ASReview's search summary figures as supplementary material (e.g. below)

We have added the progress density chart and the progress recall chart.

P5, Lines 101-4 How were these exposure categories generated? Did they come from the literature or did the authorship team create them a-priori?

Yes, as stated they were defined prior to starting the review by the review team. We mostly followed the categorizations used by our own institute in prior climate and health assessments.

P5, Lines 106-6 While the authors are to be commended for reporting their rate of disagreement, this rate is unusually high (a 28% conflict rate). How many of these were incorrect includes, and how many were incorrect excludes? Assuming all conflicts were incorrect excludes, extrapolating this rate to the large volume of articles screened at the full text stage means that as many as 397 papers are missing from the authors' analysis. This is of major concern.

Out of the 50 conflicts, 24 (48%) were incorrect excludes. However, we do not think you can directly extrapolate this to an x number of missed articles. For example, the conflict rate was higher in the requested vs randomly selected articles (~30% vs ~ 20%), and not all reviewers contributed equally to the review process. Additionally, as the review process lasted over 5 years, covered a very wide range of subjects and involved many reviewers for different parts of the process, we would expect a somewhat higher level of variation to inevitably occur. Because of the more harmonized title and abstract screening, and data clean-up and synthesis process, we are quite confident that our article selection and synthesis provides an accurate and comprehensive overview of the research field, as intended.

Results

Throughout The authors should report precise counts throughout their methods where they state results. For example, P8, Line 161 states "Most studies only quantified impacts on an exposure or generic health outcome". How many studies? It is also convention to report (n = x, X%) in all results.

We have updated the results section throughout to provide precise counts were appropriate.

The authors could also provide more concrete examples throughout their results. For example, P8 Lines 162 – 165 states "Studies on temperature or air pollution often presented the combined impacts on multiple specific health outcomes, such as cardiovascular, cerebrovascular, and respiratory diseases, while not presenting results for these outcomes individually." An example of one such study, and its result, would be helpful here.

We have added some example references here, and a number of additional descriptions of outcomes, particularly in Section 3.2.

Figures Each figure legend requires a total n represented in the figure.

Where not yet present we have added the number of publications represented in each figure.

Please specific the program used to create figures and supply the analysis source code so that this can be used in conjunction with the author's dataset to replicate their figures.

Specified under Section 2.3: “R (last used version 4.4.3) was used for data visualization.” We have provided the script for the figures in the Supplementary Materials, Appendix E. Do note that now you cannot use the full dataset provided directly, but you need to make some separate input files. With the instructions provided you should be able to replicate the figures (2-6).

They are difficult to understand in B+W. The authors might consider texture or different colours to compensate for B+W printing.

We have changed the color scale for Figure 2 to a b+w friendly scale. For the other figures the color scales should not cause issues for b+w printing and are more clear as they are then when changed to another scale.

Discussion

P11, Line 277 – 288 The authors state here that their data was used to identify gaps, however this was not a specific study aim and these gaps are absent from their results.

We have clarified in the introduction that gaps were also identified as part of the study. We have placed a bit more emphasis on the discussion of methodologies used in the results section for both retrospective and prospective research.

P12, Lines 299-312 It would be useful to highlight a specific example of how local or regional context altered the impact of adaptation and mitigation.

We have elaborated on the results of the studies already cited to provide some more insight into the possible role of adaptation and mitigation in altering the studied outcomes.

P12, Lines 313-321 This paragraph details new data that are not reported in the results. Given that this paragraph suggests the authors have additional data, it would have been an interesting addition to the results to understand the emissions projections used in each prospective study, and the modelling / prediction methods used, particularly given that the authors are aiming to describe “best practice” in this dataset.

The dataset provided in the appendix also lists the emissions/climate change scenarios used in each prospective study. We have moved this part of the discussion (+ the discussion on climate models) to Section 3.3 of the results and added additional information and a new figure on the most used scenarios and scenario combinations.

P 12, Lines 328-30 The authors could suggest a potential single methodological framework, or how one might be selected.

We have added an overview of both thematic and methodological recommendations at the end of the discussion. This can act as a sort of starting point for a general framework for future research. We have also amended the discussion where necessary to better align with these recommendations.

Minor concerns/comments

Summary

P2, Line 14 The authors could better open their summary with “Research output on the impact of climate change on human health is growing …” to better reflect the purpose of their review.

Amended as suggested.

P2, lines 18-19 The authors would be better to give precise percentages here. We now

provide precise percentages (39.2% and 31.4%)

Introduction

P4, Line 58-59 The authors could instead state that they “present a global scoping review on the impact of long-term climate change on human health” to better reflect the purpose of their review.

Amended as suggested.

Methods

P4, Line 76 Suggest changing to “Secondary impacts were defined as those that arise indirectly”

Amended as suggested.

P4, Line 78 “Tertiary effects were defined as those resulting from …” Amended as suggested.

Results

P9, Line 195 “for the exposure categories air pollution, allergens, extreme events, temperature, and water a majority of publications ..” is missing a comma after water.

Missing comma added.

Discussion

P12, Line 320-21 “For example, because it is difficult to determine what the biggest (local) impacts of climate change will be and should thus be prioritized in climate and health policies.” – this sentence does not make grammatical sense.

The sentence now reads: “Being able to make this direct quantitative comparison between outcomes is important to effectively prioritize climate change impacts in adaptation policies and actions.”

P13, Line 353-4 “such outcomes could include violent conflict or famine and was extended by us to include for example drought exposure.” – ‘was’ should be ‘were’

Amended.

Reviewer 2

The summary says that around 20% of studies looked at past impacts and 80% predicted future impacts. But it doesn’t explain how reliable these studies are. Since the review didn’t check study quality, this percentage split could be misleading.

We do not see how the differentiation between retrospective and prospective results would be affected by study quality or why splitting them based on this characteristic would be misleading. It was always clear if a study looked at the past or at the future. If the reviewer is suggesting that either retrospective or prospective studies are generally of lesser quality, and as such a quality check would disproportionately affect inclusion of studies from either group, we would be interested to hear more about this (although we have no reason to believe this was the case for our dataset).

Inclusion criteria were unclear, making it hard to distinguish weather changes from long-term climate trends, which may have caused inconsistent study selection.

There is no strict definition of long-term climate change, and as such there were some instances where this raised questions. However, for retrospective studies it often became clear from the full-text

if the authors intended to examine effects of weather or long-term climate change (e.g., because the outcome was an association between a climatic factor and a health effect, rather than an examination of a trend linked to climate change). For prospective studies it was always clear as they had to use climate change scenarios. Reviewers could request a second screening in case of doubt about such issues and those publications were screened twice.

Using ASReview in methodology section but screening only 25% of papers may be a big issue. Even though it mentions stopping rules, this could create bias, especially for less studied regions or outcomes. The sensitivity analyses is not provided in the manuscript to show how much this choice might affect results.

The models used in ASReview are trained throughout the review process using user input only (i.e., inclusions and exclusions). As we did not maintain any inclusion criteria for region or type of outcome (apart from, for health outcomes, being a primary or secondary outcome, as explained) we do not believe that this approach has biased our results towards more frequently studied regions or outcomes. We recognize that there is no fixed approach for using ASReview, and that not screening all articles will inevitably lead to missing some. To minimize this risk we used two different stopping criteria, as is generally recommended. Prior knowledge provided to ASReview's machine learning model was also different for both reviewers. Based on the large number of consecutive articles excluded before stopping, and the similar share of articles included between the first two and the third searches (2%), we do not believe that we have missed a significant number of articles (and in any case not enough to have altered our findings). We also discuss these arguments in the discussion.

Only about 11% of articles were double screened. For such a diverse topic and with AI involved; this is too low. Also, inter-rater agreement kappa score is not reported.

This was a pragmatic decision made due to resource constraints. However it was based on the methodology of other published large reviews (e.g. https://doi.org/10.1038/s43016-021-00221-z , who performed consistency checks on 10% of full texts). Our kappa score for the randomly selected full-text articles was approximately 0.45, indicating fair agreement. However, as the review process lasted over 5 years, covered a very wide range of subjects and involved many reviewers for different parts of the process, we would expect a somewhat higher level of variation to inevitably occur. Because of the more harmonized title and abstract screening, and data clean-up and synthesis process, we are quite confident that our article selection and synthesis provides an accurate and comprehensive overview of the research field, as intended.

The quality or risk of bias of included studies was not assessed. While this is sometimes acceptable in scoping reviews, here it may be a serious limitation because they extracted numerical results. This means poor-quality studies may have influenced findings just as much as stronger studies.

Yes, quality assessment is generally not a part of a scoping review. As we do not conduct any direct comparisons between the results we extracted (e.g. through meta-analysis) we do not believe this presents major issues for our analysis. However, we do agree that for further assessment (or if more diverse sources are to be included) quality controls become more important. We have now expanded on this a bit further in the discussion: "If non-peer reviewed and other grey literature had been included**, or if more direct quantitative comparisons between extracted results were made (e.g., through meta-analysis),** it would have been important to formally assess study quality." [new text in bold].

The retrospective results are mostly labeled as "variable," but the authors do not investigate why. Differences in study design, exposure definitions, or regional focus could explain this heterogeneity.

Without such analysis, the “variable” category adds little value.

In the full dataset provided in the Appendix we did list the cause(s) of variability in study outcomes for both retrospective and prospective studies. We have elaborated a bit on geographical variability in Section 3.2 with a new example, and we have added the following to the discussion: “Generally, most extracted (retrospective and prospective) climate change impact outcomes were coded as variable. This did not necessarily mean that results were inconclusive. More often it meant that we could not assign a clear direction of change to the overall outcomes within our coding framework. For example, results presented at a high spatial resolution almost always showed geographical variability. For effective climate change adaptation, results should be considered with respect to their specific local or regional context if possible.”

VERSION 2 - REVIEW

**Reviewer**	**1**
**Name**	**Fisher, Georgia**
**Affiliation**	**Macquarie University, Australian Institute of Health Innovation, Centre for Healthcare Resilience and Implementation Science**
**Date**	**11-Dec-2025**
**COI**	

I would like to thank the authorship team for their submission of their revised manuscript. While most comments have been adequately addressed, I still have concerns about the conduct of their review, and the strength of the conclusions that the authors draw.

My major concern remains around the rate of disagreement between reviewers on full-text articles. The authors response to my comment on this was relatively generic, and somewhat contradictory to their response to a similar comment from Reviewer 2. In my initial comment, my calculation of the n potential papers that were incorrectly excluded was meant for illustrative purposes only, to demonstrate the potential number of missed articles in the review, rather than an estimation of the number of papers that were indeed missed. In response, the authors states that the conflict rate was lower for randomly selected articles than those specifically requested by reviewers. However, in response to a similar comment from Reviewer 2, they also state that the kappa on double review randomly selected articles was 0.45. This is still too low to provide assurance that the authors have not missed a substantial amount of the relevant literature in their review, which, as they report counts and percentages, and use these to make conclusions and recommendations, is of concern. Also of concern is the absence of mention of this methodological choice in their description of study limitations, where only the limitations of the title abstract screening were discussed.

The authors could address these concerns by conducting additional screening on the ~350 papers that were excluded at full text, to at least ensure that reviewer(s) did not consistently exclude a

particular type of paper, and bias the dataset. At a bare minimum, this possibility should have been discussed as a study limitation.

My second ongoing concern pertains to the accuracy of the dataset and figures created by the authors.

When I ran my (own, and then AI confirmed) check of the number of unique paper titles included in the authors raw dataset, I got 1213 papers (rather than 1215). On further inspection, there are two duplicate titles with different ID numbers in the dataset provided:

1. Climate Change and the Risk of Malaria Transmission in Iran (A. A. Hanafi-Bojd)

2. Increased high-temperature extremes and associated population exposure in Africa by the mid-21st century, (Iyakaremye, V.)

Again, in my (own, and then AI confirmed) checks, four papers in the dataset do not list a region (N/A in sheet), and thus Figure 2b shows data from 1211 papers, rather than the 1215 as the legend specifies.

These errors should be rectified in study analyses, and do raise concerns about potential further errors in data and figures.

Finally, although not as consequential:

I also ran a spot check (AI led) of Figure 2b code, where the following flag was raised.

separate_rows(exp, sep = ";") and separate_rows(region, sep = ";") produce Cartesian expansion. If a paper has 2 exposures and 3 regions, it yields 6 rows before counting—each counted as a separate paper. The y-axis label says "Number of papers", but the chart actually shows paper-exposure-region combinations, not unique papers. This could inflate counts and mislead, particularly as the figure legend states that a paper was only counted once for a particular exposure category.

As I am not an R expert and note that the authors provided extra data cleaning instructions, I only suggest that the authors check that this flag does not affect their figure. This issue did not form a part of my decision on this paper.

VERSION 2 - AUTHOR RESPONSE

Reviewer 1

I would like to thank the authorship team for their submission of their revised manuscript. While most comments have been adequately addressed, I still have concerns about the conduct of their review, and the strength of the conclusions that the authors draw.

My major concern remains around the rate of disagreement between reviewers on full-text articles. The authors response to my comment on this was relatively generic, and somewhat contradictory to their response to a similar comment from Reviewer 2. In my initial comment, my calculation of the n potential papers that were incorrectly excluded was meant for illustrative purposes only, to demonstrate the potential number of missed articles in the review, rather than an estimation of the number of papers that were indeed missed. In response, the authors states that the conflict rate was

lower for randomly selected articles than those specifically requested by reviewers. However, in response to a similar comment from Reviewer 2, they also state that the kappa on double review randomly selected articles was 0.45. This is still too low to provide assurance that the authors have not missed a substantial amount of the relevant literature in their review, which, as they report counts and percentages, and use these to make conclusions and recommendations, is of concern. Also of concern is the absence of mention of this methodological choice in their description of study limitations, where only the limitations of the title abstract screening were discussed.

The authors could address these concerns by conducting additional screening on the ~350 papers that were excluded at full text, to at least ensure that reviewer(s) did not consistently exclude a particular type of paper, and bias the dataset. At a bare minimum, this possibility should have been discussed as a study limitation.

We thank the reviewer for their careful assessment of our manuscript and dataset. The lead reviewer (JL) has screened again all papers that were initially excluded by another reviewer (187/363 initially excluded papers). This has yielded 10 new inclusions. Based on this limited disagreement we remain confident that our approach was appropriate, especially considering the scope of the work. For reference we have included at the end of this document a table containing all exclusion decisions, highlighting the papers that have been included following this second screening. We have updated the methodology to reflect this additional step, and have updated text and figures accordingly. We have also included this limitation as a discussion point. We hope that this sufficiently addresses the reviewer's concerns.

My second ongoing concern pertains to the accuracy of the dataset and figures created by the authors.

When I ran my (own, and then AI confirmed) check of the number of unique paper titles included in the authors raw dataset, I got 1213 papers (rather than 1215). On further inspection, there are two duplicate titles with different ID numbers in the dataset provided:

1. Climate Change and the Risk of Malaria Transmission in Iran (A. A. Hanafi-Bojd)

2. Increased high-temperature extremes and associated population exposure in Africa by the mid-21st century, (Iyakaremye, V.)

We have removed these duplicates from the dataset. We have confirmed no other duplicates remain.

Again, in my (own, and then AI confirmed) checks, four papers in the dataset do not list a region (N/A in sheet), and thus Figure 2b shows data from 1211 papers, rather than the 1215 as the legend specifies.

We have amended captions, text and figures based on the additional screening with consideration of this remark.

These errors should be rectified in study analyses, and do raise concerns about potential further errors in data and figures.

We have checked the text for any remaining inconsistencies or errors, including those remaining from previous versions of the manuscript, and have made minimal amendments were needed.

Finally, although not as consequential:

I also ran a spot check (AI led) of Figure 2b code, where the following flag was raised.

separate_rows(exp, sep = ";") and separate_rows(region, sep = ";") produce Cartesian expansion. If a paper has 2 exposures and 3 regions, it yields 6 rows before counting—each counted as a separate

paper. The y-axis label says “Number of papers”, but the chart actually shows paper-exposure-region combinations, not unique papers. This could inflate counts and mislead, particularly as the figure legend states that a paper was only counted once for a particular exposure category.

As I am not an R expert and note that the authors provided extra data cleaning instructions, I only suggest that the authors check that this flag does not affect their figure. This issue did not form a part of my decision on this paper.

Yes, this assessment is correct. If a paper studies 2 main exposure categories across 3 regions, it should be represented by 6 ‘entries’ in the figure. We have added some clarification to the caption, stating that one paper can be counted multiple times per region.
