# Supplementary material for: Impacts of long-term climate change on human health: a global scoping review of quantitative research
Source: BMJ Open. 2026 Sep 30;16(9):e108532. doi: 10.1136/bmjopen-2025-108532 (PMC13630005; doi:10.1136/bmjopen-2025-108532)
Supplement: Supplementary file 2 [file bmjopen-16-9-s001.docx]

**Supplementary materials to “Impacts of long-term climate change on human health: a global scoping review of quantitative research”**

Jesse Limaheluw, Rebecca Niese, Dieneke Bijkerk-Schram, Lisbeth Hall, Jelske de Kraker, Saskia Oostdijk, Fábio Serafim, Ana Maria de Roda Husman

**Appendix A – Full search query p.2**

**Appendix B – Data extraction outcomes p.3**

**Appendix C – Additional figures p.4**

**Appendix D – ASReview output figures p.5**

**Appendix E – R scripts used to make figures p.6**

**Appendix A – Full search query**

*Embase*

(‘climate change’/exp OR ‘climat* change’:ti,ab,kw OR ‘greenhouse effect’/exp OR ‘greenhouse effect’:ti,ab,kw OR ‘global warming’:ti,ab,kw OR ‘climat* sensitivity’:ti,ab,kw OR ‘climat* variability’:ti,ab,kw)

AND

('health'/exp OR 'health':ti,ab,kw OR 'wellbeing'/exp OR 'wellbeing':ti,ab,kw OR ‘well-being’:ti,ab,kw OR 'morbidity'/exp OR 'morbidity':ti,ab,kw OR 'mortality'/exp OR 'mortality':ti,ab,kw OR 'diseases'/exp OR 'disease?':ti,ab,kw)

Climate change, greenhouse effect, health, wellbeing, morbidity, mortality, and diseases are included as Emtree terms.

*PubMed*

(“climate change”[MeSH] OR “climate change”[Text Word] OR “climatic change”[Text Word] OR “climate”[MeSH:noexp] OR “greenhouse effect”[MeSH] OR “greenhouse effect”[Text Word] OR “global warming”[Text Word] OR “climate variability”[Text Word] OR “climatic variability”[Text Word] OR “climate sensitivity”[Text Word] OR “climatic sensitivity”[Text Word])

AND

(“health”[MeSH] OR “health”[Text Word] OR “wellbeing”[Text Word] OR “well-being”[Text Word] OR “morbidity”[MeSH] OR “morbidity”[Text Word] OR “mortality”[MeSH] OR “mortality”[Text Word] OR “disease”[MeSH] OR “disease”[Text Word] OR “diseases”[Text Word])

Climate change, climate, greenhouse effect, health, morbidity, mortality and disease are included as MeSH terms. NB. “climate change” was introduced as a MeSH term in 2010, before 2010 articles on this subject were indexed under the “climate” MeSH

*Scopus*

TITLE-ABS (“climat* change” OR “greenhouse effect” OR “global warming” OR “climat* variability” OR “climat* sensitivity”)

AND

TITLE-ABS (health OR wellbeing OR “well-being” OR morbidity OR mortality OR disease)

Limits

Published in or after 2000, in English. Included document types are journal articles (including in-press) and reviews.

**Appendix B – Data extraction outcomes**

Access the data extraction file “limaheluw et al – appendix b - data extraction outcomes - withID” through the following link: <https://docs.google.com/spreadsheets/d/1cbBGMXBIAp9IB-81Q6_nRM69z_q-kA5wZJwyvzgkTYg/edit?usp=sharing>

To enable filtering, select data range A11: S2232 and go to Data > Create filter view.

**Appendix C – Additional figures**

***Figure C1*** *Number of times each year between 1950 and 2100 was represented in retrospective (blue) and prospective (green) studies. If study results represented (the average of) a time period, e.g., 2041-2060, all years within that time period were counted for this figure.*

***Figure C2*** *Distribution of main outcome types per exposure category. We only counted an exposure category once per paper. When a publication examined multiple exposures in the same category with different outcome types, all outcome types were counted.*

**Appendix D – ASReview output figures**

***Figure D1*** *Progress density chart.*

***Figure D2*** *Progress recall chart.*

**Appendix E – R scripts used to make figures**

\## All figures

library(dplyr);library(tidyr);library(ggplot2);library(pals);library(patchwork);library(ComplexUpset);library(stringr);library(cowplot);library(ggpubr)

\## Input files

\# for figure 2

\# Input csv needs the have at least the following columns: 'exp'(= main exposure category), 'region'(= WHO region), and 'year'(= publication year)

\# in combined format (i.e., one row per paper, with different exposures, regions etc. separated by semicolon)

base.out <- read.csv2("base_output_csv.csv")

\# for figure 3-5

\# Input csv needs to have a long format (i.e. full dataset can be used as provided) with at least the following columns:

\# 'type'(= 'Retrospective' or 'Prospective'), 'exp_1'(= main exposure category), 'exp_2'(= secondary exposure category), 'gbd_1'(= primary gbd category [health outcomes only]),

\# 'gbd_3'(= tertiary gbd category), 'impact' (= increase/decrease/variable/no (significant) effect), 'exposure_only'(= yes/no),

\# 'health_outcome'(= yes/no > combinded column for generic and specific impacts)

outcomes.full <- read.csv2("outcomes_full_up.csv")

\# for figure 6

\# Note: compatibility issues with recent ggplot2 version (4.0.0), use ggplot2 version 3.5.2 until fixed

\# Input csv needs to have only column names corresponding to the different scenarios you want to plot (here, RCP1.9-RCP8.5)

\# each row is a publication and the included scenario names are specified in each corresponding column

upset.data <- read.table("Inputs/upset_rcps.csv", na.strings = c("", NA), sep = ";",

header = T)

\## Figure 2

\# Figure 2a

\# Count times exposures studied per year

exp.counted <- base.out %>%

separate_rows(exp, sep = ";") %>%

mutate(exp = str_trim(exp)) %>%

mutate(exp = na_if(exp, "NA")) %>%

count(year, exp)

exp.counted$exp[exp.counted$exp == "Other"] <- "y"

exp.counted$exp[is.na(exp.counted$exp)] <- "z"

years <- c(2000:2024)

years.lab <- c(2000:2023, "2024 up to May")

(count.exp <- ggplot(data = exp.counted, aes(x = year, y = n, fill = exp)) +

geom_bar(position = "stack", stat = "identity", color = "black") +

xlab("Year") + ylab("Number of papers") +

theme_classic() +

theme(panel.border = element_rect(color = "black", fill = NA),

axis.text = element_text(size = 14),

axis.title = element_text(size = 15),

legend.text = element_text(size = 14, margin = margin(l = 5, r = 15)),

legend.title = element_blank(),

legend.spacing.x = unit(1.5, "cm"),

legend.position = "bottom") +

guides(fill = guide_legend(nrow = 4, by.row = TRUE)) +

#labs(fill = "Health exposure category") +

scale_fill_viridis_d(labels = c("Air pollution", "Allergens", "Extreme events", "Food", "Soil", "Temperature", "UV", "Vectors and other animals", "Water", "Other", "Unspecified")) +

scale_x_continuous(labels = years.lab, breaks = years) +

coord_flip())

\# Figure 2b

\# Count times exposure studies per region

exp.counted.reg <- base.out %>%

separate_rows(exp, sep = ";") %>%

mutate(exp = str_trim(exp)) %>%

separate_rows(region, sep = ";") %>%

mutate(exp = na_if(exp, "NA")) %>%

count(region, exp) %>%

drop_na(region)

exp.counted.reg$exp[exp.counted.reg$exp == "Other"] <- "y"

exp.counted.reg$exp[is.na(exp.counted.reg$exp)] <- "z"

(count.expreg <- ggplot(data = exp.counted.reg, aes(x = region, y = n, fill = exp)) +

geom_bar(position = "stack", stat = "identity", color = "black") +

xlab("WHO region") + ylab("Number of papers") +

theme_classic() +

theme(panel.border = element_rect(color = "black", fill = NA),

axis.text = element_text(size = 14),

axis.title = element_text(size = 15),

legend.text = element_text(size = 14, margin = margin(l = 5, r = 15)),

legend.title = element_blank(),

legend.spacing.x = unit(1.5, "cm"),

legend.position = "bottom") +

guides(fill = guide_legend(nrow = 4, by.row = TRUE)) +

#labs(fill = "Health exposure category") +

scale_fill_viridis_d(labels = c("Air pollution", "Allergens", "Extreme events", "Food", "Soil", "Temperature", "UV", "Vectors and other animals", "Water", "Other", "Unspecified")) +

coord_flip())

\# arrange figures 2a and b

common.legend <- get_legend(count.expreg)

arranged <- ggarrange(count.exp, count.expreg, ncol = 1, nrow = 2,

labels = "AUTO", legend.grob = common.legend, legend = "bottom", align = "h")

\# save figure 2

ggsave("Output/fig2-update3.png", plot = arranged, height = 15, width = 10, dpi = 300)

\## figure 3

\# Load data

health.all <- outcomes.full %>%

filter(specific_health == "yes")

gbd13 <- data.frame(health.all$gbd1, health.all$gbd3)

gbd13 <- gbd13[!duplicated(gbd13), ]

colnames(gbd13) <- c("gbd1", "gbd3")

#Split rows

health.split <- health.all %>%

separate_rows(exp_1, sep = ";") %>%

mutate(exp_1 = str_trim(exp_1)) #%>%

#separate_rows(region, sep = ";") %>%

#mutate(region = str_trim(region))

#For plot by exposure

outcomesbyexposure.count <- health.split %>%

count(gbd3, exp_1) %>%

na.omit()

outcomesbyexposure.count <- inner_join(outcomesbyexposure.count, gbd13, by = "gbd3")

\# Add sum row

obe.count.ag <- outcomesbyexposure.count %>%

group_by(gbd3, gbd1) %>%

summarise(exp_1 = "Total",

n = sum(n))

outcomesbyexposure.count <- rbind(outcomesbyexposure.count, obe.count.ag)

expcount.plot <- outcomesbyexposure.count %>%

mutate(exp_1_rec = recode(exp_1,

"Air pollution (excl. allergens)" = "b",

"Allergens" = "c",

"Extreme events (excl. heat- and coldwaves)" = "d",

"Food" = "e",

"Soil" = "f",

"Temperature (incl. heat- and coldwaves)" = "g",

"UV" = "h",

"Vectors and other animals" = "i",

"Water" = "j",

"Other" = "k",

"N/A" = "l",

"Total" = "a")) %>%

mutate(y_cat = recode(exp_1_rec,

"a" = "t",

"b" = "s",

"c" = "s",

"d" = "s",

"e" = "s",

"f" = "s",

"g" = "s",

"h" = "s",

"i" = "s",

"j" = "s",

"k" = "s",

"l" = "s"))

outcome_labs <- c("Total", "Air pollution", "Allergens", "Extreme events", "Food",

"Soil", "Temperature", "UV", "Vectors and other animals", "Water",

"Other", "Unspecified")

#Plot by exposure

(heat.exp <- ggplot(data = expcount.plot, aes(x = exp_1_rec, y = gbd3)) +

geom_tile(aes(fill = n), color = "white") +

scale_fill_gradientn(colors = kovesi.isoluminant_cgo_70_c39(8)) +

#scale_fill_gradient(low = "lightblue", high = "darkblue") +

geom_text(aes(label = n)) +

#facet_grid(rows = vars(expcount.plot$gbd1), scales = "free", space = "free",

\#	labeller = labeller(gbd1 = gbd.labels)) +

facet_grid(cols = vars(expcount.plot$y_cat),

rows = vars(expcount.plot$gbd1), scales = "free", space = "free",

labeller = labeller(gbd1 = as_labeller(c("Communicable, maternal, neonatal, or nutritional (CMNN) diseases", "Injuries", "Non-communicable disease")))) +

scale_x_discrete(breaks = levels(factor(expcount.plot$exp_1_rec)),

labels = outcome_labs) +

scale_y_discrete(limits = rev) +

ylab("Global Burden of Disease level 3 outcomes") +

xlab("Exposure category") +

theme(axis.text.x = element_text(angle = 45, hjust = 1, size = 12),

axis.text.y = element_text(size = 12),

axis.title = element_text(size = 13),

strip.text.x = element_blank(),

strip.text.y = element_text(size = 11)) +

labs(fill = "Count"))

ggsave("Output/figure3_update3.png", plot = heat.exp, width = 10, height = 19, dpi = 300)

\## figure 4

\# Barcharts exposure and generic health outcomes

ret.exp <- outcomes.full %>%

filter(grepl("Retrospective", type)) %>%

filter(grepl("yes", exposure_only)) %>%

separate_rows(exp_1, exp_2, sep = ";") %>%

mutate(exp_1 = str_trim(exp_1)) %>%

count(impact, exp_1)

ret.exp$exp_1[ret.exp$exp_1 == "Other"] <- "y"

#unsp <- data.frame("Increase", "z", 0)

#names(unsp) <- names(ret.exp)

#ret.exp <- rbind(ret.exp, unsp)

(exp.only.r <- ggplot(data = ret.exp, aes(x = exp_1, y = n, fill = impact)) +

geom_bar(position = "stack", stat = "identity") +

xlab("Exposure category") + ylab("Count") +

theme_classic() +

theme(panel.border = element_rect(color = "black", fill = NA),

axis.text = element_text(size = 16),

axis.title = element_text(size = 16),

legend.text = element_text(size = 16),

legend.title = element_blank(),

legend.position = "bottom",

plot.title = element_text(size = 20)) +

scale_fill_manual(values = c("#2374AB", "#FF8484", "#C8C8C8","#EDD892")) +

scale_x_discrete(limits = rev,

labels = rev(c("Air pollution", "Allergens", "Extreme events", "Temperature",

"UV", "Vectors and other animals", "Water", "Other"))) +

coord_flip() +

ggtitle("Exposure only"))

#ggsave("exp_only_retrospective_update2.png", plot = exp.only)

\# all health outcomes together

ret.gen <- outcomes.full %>%

filter(grepl("Retrospective", type)) %>%

separate_rows(exp_1, sep = ";") %>%

separate_rows(exp_2, sep = ";") %>%

mutate(exp_1 = str_trim(exp_1)) %>%

count(impact, exp_1, health_outcome)

ret.gen$n[ret.gen$health_outcome == "no"] <- 0

ret.gen$exp_1[ret.gen$exp_1 == "Other"] <- "y"

ret.gen$exp_1[ret.gen$exp_1 == "N/A"] <- "z"

(health.only.r <- ggplot(data = ret.gen, aes(x = exp_1, y = n, fill = impact)) +

geom_bar(position = "stack", stat = "identity") +

xlab("Exposure category") + ylab("Count") +

theme_classic() +

theme(panel.border = element_rect(color = "black", fill = NA),

axis.text = element_text(size = 16),

axis.title = element_text(size = 16),

legend.text = element_text(size = 16),

legend.title = element_blank(),

legend.position = "bottom",

plot.title = element_text(size = 20)) +

scale_fill_manual(values = c("#2374AB", "#FF8484", "#C8C8C8","#EDD892")) +

scale_x_discrete(limits = rev,

labels = rev(c("Air pollution", "Allergens", "Extreme events", "Temperature",

"UV", "Vectors and other animals", "Water", "Other"))) +

coord_flip() +

ggtitle("Health outcomes (generic + specific)"))

#ggsave("health_outcomes_retrospective_update2.tiff", plot = gen.only)

patch1 <- (exp.only.r | health.only.r) + plot_layout(guides = "collect") & theme(legend.position = "bottom")

patch1[[2]] <- patch1[[2]] + theme(axis.text.y = element_blank(),

axis.ticks.y = element_blank(),

axis.title.y = element_blank())

patch1

ggsave("Output/fig_4retrospective_outcomes_update3.png", plot = patch1, width = 17.5, height = 7.5, dpi = 300)

\## figure 5

pro.exp <- outcomes.full %>%

filter(grepl("Prospective", type)) %>%

filter(grepl("yes", exposure_only)) %>%

separate_rows(exp_1, exp_2, sep = ";") %>%

mutate(exp_1 = str_trim(exp_1)) %>%

count(impact, exp_1)

pro.exp$exp_1[pro.exp$exp_1 == "Other"] <- "y"

pro.exp$exp_1[pro.exp$exp_1 == "N/A"] <- "z"

(exp.only.p <- ggplot(data = pro.exp, aes(x = exp_1, y = n, fill = impact)) +

geom_bar(position = "stack", stat = "identity") +

xlab("Exposure category") + ylab("Count") +

theme_classic() +

theme(panel.border = element_rect(color = "black", fill = NA),

axis.text = element_text(size = 16),

axis.title = element_text(size = 16),

legend.text = element_text(size = 16),

legend.title = element_blank(),

legend.position = "bottom",

plot.title = element_text(size = 20)) +

scale_fill_manual(values = c("#2374AB", "#FF8484", "#C8C8C8","#EDD892")) +

scale_x_discrete(limits = rev,

labels = rev(c("Air pollution", "Allergens", "Extreme events", "Food", "Soil", "Temperature",

"UV", "Vectors and other animals", "Water", "Other", "Unspecified"))) +

coord_flip() +

ggtitle("Exposure only"))

#ggsave("exp_only_retrospective_update2.tiff", plot = exp.only)

\# all health outcomes together

pro.gen <- outcomes.full %>%

filter(grepl("Prospective", type)) %>%

separate_rows(exp_1, sep = ";") %>%

separate_rows(exp_2, sep = ";") %>%

mutate(exp_1 = str_trim(exp_1)) %>%

count(impact, exp_1, health_outcome)

pro.gen$n[pro.gen$health_outcome == "no"] <- 0

pro.gen$exp_1[pro.gen$exp_1 == "Other"] <- "y"

pro.gen$exp_1[pro.gen$exp_1 == "N/A"] <- "z"

(health.only.p <- ggplot(data = pro.gen, aes(x = exp_1, y = n, fill = impact)) +

geom_bar(position = "stack", stat = "identity") +

xlab("Exposure category") + ylab("Count") +

theme_classic() +

theme(panel.border = element_rect(color = "black", fill = NA),

axis.text = element_text(size = 16),

axis.title = element_text(size = 16),

legend.text = element_text(size = 16),

legend.title = element_blank(),

legend.position = "bottom",

plot.title = element_text(size = 20)) +

scale_fill_manual(values = c("#2374AB", "#FF8484", "#C8C8C8","#EDD892")) +

scale_x_discrete(limits = rev,

labels = rev(c("Air pollution", "Allergens", "Extreme events", "Food", "Soil", "Temperature",

"UV", "Vectors and other animals", "Water", "Other", "Unspecified"))) +

coord_flip() +

ggtitle("Health outcomes (generic + specific)"))

#ggsave("health_outcomes_retrospective_update2.tiff", plot = gen.only)

patch1 <- (exp.only.p | health.only.p) + plot_layout(guides = "collect") & theme(legend.position = "bottom")

patch1[[2]] <- patch1[[2]] + theme(axis.text.y = element_blank(),

axis.ticks.y = element_blank(),

axis.title.y = element_blank())

#patch1

ggsave("Output/fig5_prospective_outcomes_update2.png", plot = patch1, width = 17.5, height = 7.5, dpi = 300)

\## figure 6

unique.vals <- upset.data %>%

dplyr::count(RCP1.9, RCP2.6, RCP4.5, RCP6, RCP7, RCP8.5)

unique.vals2 <- unique.vals

unique.vals2[!is.na(unique.vals2)] <- TRUE

unique.vals2[is.na(unique.vals2)] <- FALSE

unique.vals2$n <- unique.vals$n

overall <- uncount(unique.vals2, n)

colnames(overall) <- c("RCP1.9", "RCP2.6", "RCP4.5", "RCP6", "RCP7", "RCP8.5")

cols <- colnames(overall)

upset.plot <- upset(data = overall, intersect = cols,

name = "RCP combinations",

base_annotations = list(

"RCP combination counts" = intersection_size(counts = FALSE)

),

set_sizes = upset_set_size(position = "right"),

min_size = 5)

ggsave("Output/fig6_upset_plot3.png", plot = upset.plot, width = 10, dpi = 300)
